# Supplementary material for: ‘Because my brain isn’t as active as it should be, my eyes don’t always see’: a qualitative exploration of the stress process for those living with posterior cortical atrophy
Source: BMJ Open. 2018 Feb 8;8(2):e018663. doi: 10.1136/bmjopen-2017-018663 (PMC5829605; doi:10.1136/bmjopen-2017-018663)
Supplement: Author's manuscript [file bmjopen-2017-018663.draft_revisions.pdf]

BMJ Open

BMJ Open is committed to open peer review. As part of this commitment we make the peer review history of every article we publish publicly available.

When an article is published we post the peer reviewers' comments and the authors' responses online. We also post the versions of the paper that were used during peer review. These are the versions that the peer review comments apply to.

The versions of the paper that follow are the versions that were submitted during the peer review process. They are not the versions of record or the final published versions. They should not be cited or distributed as the published version of this manuscript.

BMJ Open is an open access journal and the full, final, typeset and author-corrected version of record of the manuscript is available on our site with no access controls, subscription charges or pay-per-view fees (http://bmjopen.bmj.com).

If you have any questions on BMJ Open's open peer review process please email editorial.bmjopen@bmj.com

BMJ Open

‘Because my brain isn’t as active as it should be, my eyes don’t always see’ – a qualitative exploration of the stress process for those living with posterior cortical atrophy

Journal:	*BMJ Open*
Manuscript ID	bmjopen-2017-018663
Article Type:	Research
Date Submitted by the Author:	13-Jul-2017

Complete List of Authors:	Harding, Emma; University College London Institute of Neurology, Dementia Research Centre<br>Sullivan, Mary Pat; Nipissing University, School of Human and Social Development; University College London Institute of Neurology, Dementia Research Centre<br>Woodbridge, Rachel; Brunel University, Department of Clinical Sciences<br>Yong, Keir; University College London Institute of Neurology, Dementia Research Centre<br>McIntyre, Anne; Brunel University, Department of Clinical Sciences<br>Gilhooly, Mary; Brunel University, School of Health Science and Social Care<br>Gilhooly, KJ; Brunel University, Department of Clinical Sciences<br>Crutch, Sebastian; University College London Institute of Neurology

**Primary Subject Heading**:	Qualitative research
Secondary Subject Heading:	Neurology
Keywords:	Dementia < NEUROLOGY, EDUCATION & TRAINING (see Medical Education & Training), Neuro-ophthalmology < NEUROLOGY, PRIMARY CARE, QUALITATIVE RESEARCH

SCHOLARONE™ Manuscripts

‘Because my brain isn’t as active as it should be, my eyes don’t always see’ – a qualitative exploration of the stress process for those living with posterior cortical atrophy

Word count: 6943

Keywords: [MeSH terms] Alzheimer Disease, Dementia, Vision Disorders, Family, Qualitative Resear

Authors and affiliations

Emma Harding[1] BSc., Mary Pat Sullivan[2] PhD., Rachel Woodbridge[3] BSc., Keir Yong[1] PhD., Anne McIntyre[3] PhD., Mary L. Gilhooly[3] PhD., Kenneth J. Gilhooly[3] PhD., Sebastian J. Crutch[1] PhD.

[1.] Dementia Research Centre, University College London, London, UK

[2.] Faculty of Applied and Professional Studies, Nipissing University, North Bay, Canada

[3.] Department of Clinical Sciences, Brunel University London, Uxbridge, UK

Correspondence to:

Emma Harding

Box 16, National Hospital for Neurology and Neurosurgery

Queen Square

London, WC1N 3BG

emma.harding@ucl.ac.uk

020 3488 3667 (Mondays and Fridays)

1. Abstract

Objectives: To explore the stress process for individuals living with posterior cortical atrophy (PCA) and their families.

Design: A qualitative study using in-depth semi-structured dyadic and individual interviews with people living with a diagnosis of PCA and a family carer. Interview transcripts were thematically analysed.

Setting: Participants’ homes.

Participants: 20 individuals in the mild to moderate stages of PCA and 20 family carers.

For peer review only

Findings: Three major themes were identified – (1) The diagnostic journey: mostly an unsettling and convoluted process, owing to the early age of onset, rarity and atypical symptom profile of PCA. (2) Interactions with the physical environment: profound difficulties with functional and leisure activities were usually compensated for with adaptations maximising familiarity or simplicity. (3) Implications within the psychosocial environment: symptoms impacted individuals' sense of independence and identity and required reallocations of roles and responsibilities. Ongoing uncertainties and the progressive nature of PCA caused most dyads to take a 'one day at a time' approach to coping. Relatively well-preserved insight and memory were a benefit and burden, as individuals shared the illness experience with family members but also compared their current situation to before diagnosis. The experience was framed by background and contextual factors and understood within an ever-changing temporal context.

Conclusion: The stress process in PCA is characterised by uncertainty and unpredictability from diagnosis through to ongoing management. The provision of tailored information about cortical visual problems and associated functional difficulties, time-sensitive environmental adaptations to help those with PCA to identify what and where things are, and psychosocial interventions for the marital/family unit as a whole would be useful to improve both functional status and psychological wellbeing. Future research exploring (i) stress and coping in the later stages of PCA and (ii) the nature and impact of visual impairment(s) in typical Alzheimer's disease would be worthwhile.

2. Strengths and limitations of this study

As the first qualitative study of those living with PCA this paper provides original, in-depth insights into the subjective experiences of those with dementia-related visual impairment.

As well as providing empirical description of the illness experience, in using a conceptual framework (the Stress Process Model) this study also makes a broader contribution to social science and the field of dementia research.

With both individual and dyadic interviews, this study allowed the multiple perspectives of both people with the diagnosis and their family carer – individual and shared narratives – to emerge.

Conducting interviews in participants' homes permitted a rich understanding of the physical and psychosocial context within which daily difficulties owing to visual processing problems emerged.

Despite the progressive nature of PCA the interviews were conducted at one point in time so any insights gleaned about the nature of the disease progression and associated stress relied on

For peer review only - http://bmjopen.bmj.com/site/about/guidelines.xhtml

participants' abilities to recall prior events. Future longitudinal research would be valuable to develop the current study findings.

3. Background

There are an estimated 850,000 people currently living with dementia in the UK (Alzheimer's Society, 2014) and it is estimated that 5% of these (approx. 42,500) are cases of young onset dementias, with symptoms beginning before the age of 65 (Alzheimer's Society, 2014). Posterior Cortical Atrophy, originally called Benson's disease, is a rare form of dementia which is typically early in onset with symptoms usually beginning between the ages of 50 and 65 years (Crutch et al., 2017). The underlying pathology in the majority of individuals is Alzheimer's disease (AD) although a small number of cases attributable to Lewy Body disease and corticobasal degeneration have been reported (e.g. Tang-Wai et al., 2004; Renner et al., 2004). The prevalence is unknown. PCA is characterised by initial neurodegeneration towards the back of the brain, specifically in the parietal, occipital, and occipitotemporal regions (Crutch et al., 2012). Correspondingly, the initial symptoms predominantly relate to cortical visual impairment, particularly deficits in visuo-spatial and visuo-perceptual processing. Other symptoms include impairments in literacy, spelling and numeracy, but contrary to more typical amnestic presentations of AD, episodic memory functions are relatively well preserved, at least in the early stages of PCA.

When compared to other dementias PCA is relatively under-researched. The majority of research into PCA is concentrated around establishing the neuropsychological, cognitive and imaging profile(s) of those with the diagnosis (e.g. Victoroff et al., 1994; Mendez, Ghajarania & Perryman, 2002; Tang-Wai et al., 2004; Lehmann et al., 2011). Anecdotal and laboratory based evidence suggests ways in which PCA might impact on people's daily lives, including problems with reading, driving and localising objects in space (Mendez, Ghajarania & Perryman, 2002; Charles & Hillis 2005; Yong et al., 2014), but there is a paucity of research focusing on the everyday impact of living with the diagnosis for individuals and families.

One recent paper investigated the impact of PCA on activities of daily living, documenting difficulties with performance of everyday skills for people with PCA (including operation of appliances, writing and handling money) and self-care (including dressing, feeding and bathing) compared to predominant deficits in memory, motivation and orientation of people with typical, amnestic presentations of Alzheimer's disease (tAD) (Shakespeare et al., 2015). In another recent paper, Suárez-González and colleagues (2016) investigated the neuropsychiatric profile of people with PCA.

For peer review only - http://bmjopen.bmj.com/site/about/guidelines.xhtml

They found similar increases in depression, apathy, irritability and anxiety for those with PCA to participants with tAD.

In the absence of disease modifying therapies for Alzheimer's disease and other forms of dementia, environmental and psychosocial interventions to improve the quality of life and wellbeing of those living with it hold particular significance (e.g. Kasl-Godley & Gatz, 2000; Cahill et al., 2008; Gilhooly et al., 2016). Better understanding the needs of those with different rare and/or young onset dementias will be an important step in developing effective environmental or psychosocial interventions. A second generation of literature is beginning to delineate the particular experiences of those with less common forms of dementias such as young onset dementias (e.g. Bakker et al., 2010; Johannessen & Möller, 2013) and those with atypical symptom profiles like behavioural variant frontotemporal dementia (e.g. Griffin, Oyebode & Allen, 2016). Developing a greater understanding of the day-to-day impact of dementia in relation to visual problems will be a timely addition to this, not least because those with typical amnestic presentations of AD may also go on to have cortical visual impairment, likely later on in their diagnosis and as such at a time when they may not be so able to articulate their experiences of the symptoms (e.g. Paxton et al., 2007).

The current study sought to maximise on the abilities of those with PCA to reflect on and communicate their experiences. Using the Stress Process Model (Pearlin et al., 1990; Judge et al., 2010) as a conceptual framework we present findings from a qualitative exploration of the stresses associated with mild to moderate stage PCA and responses to these over time. The Stress Process Model outlines primary stressors which result directly from the disease itself, secondary strains which may follow, and both internal and external factors which mediate both of these in shaping outcomes. Having been developed to conceptualise informal caregivers' experiences (Pearlin et al., 1990), it has since been adapted for individuals with dementia (Judge et al., 2010), and in doing so acknowledges the multiple perspectives to be taken into account when understanding the dementia experience. More specifically, the study aimed to explore the potential of the physical environment to contribute to and/or mediate the stress process owing to the prominent visuo-spatial and visuo-perceptual symptoms.

4. Methods

Design/sampling

A qualitative design was deemed appropriate in order to gain rich detailed accounts and for possibly unanticipated insights to emerge (Rubin & Rubin, 2011). This was considered important because of both the paucity of knowledge about the impact of PCA on individuals' subjective experiences and

For peer review only - http://bmjopen.bmj.com/site/about/guidelines.xhtml

also the distinctiveness of the symptom profile. In-depth semi-structured interviews were chosen owing to the abilities of those with PCA to recount their experiences and again, to facilitate the collection of data of sufficient richness and depth. Participants were recruited via the Specialist Cognitive Disorders Clinic at the National Hospital for Neurology and Neurosurgery, University College London Hospitals NHS Foundation Trust. Inclusion criteria were a confirmed diagnosis of PCA and an accompanying family member or familiar other also willing to participate.

Twenty individuals with PCA (12 F; 8 M) took part in the interviews and the mean age was 68 years (7.66 SD). Scores on the Mini Mental State Examination (MMSE) ranged from 10-29 (mean=20.05; SD=6.54), indicating mild to moderate dementia. Twenty spouses/family carers took part (10 F; 10 M); in 18 cases this was a spouse, in one case the dyadic relationship was mother-daughter and in the other case it was aunt-niece. One spouse (F) opted not to take part in an individual interview, but did participate in the dyadic one. Only one participant lived alone. The number of years since diagnosis ranged from 0 to 12 (mean=3.31; SD=2.75) and the number of years since subjective onset ranged from 2 to 14 (mean=6.39; SD=3.26).

A comparative sample of people living with typical Alzheimer's disease (n=17) and their family carers (n=17) were also interviewed and findings from the subsequent analysis of that data will be reported in another paper.

Ethical approval

The study was approved by the National Research Ethics Service Committee – London Queen Square and informed consent was obtained from all participants. After the interviews the researchers conducted a short debrief providing further information and contact details in case of any issues or causes for concern related to the study.

Data collection

Individual and dyadic interviews were conducted at participants' homes. Dyads were interviewed together and then separately in order to capture the dyads' shared experience (Taylor & de Vocht, 2011) and provide the opportunity for individuals to provide information they might not feel comfortable to disclose in the presence of their family member (Morgan et al., 2013). The interview schedule covered contextual factors (personal, marital and occupational history, current family situation), the diagnostic journey, and daily difficulties and coping strategies within the home environment. In total interviews lasted between 3-4 hours per dyad. The home visit also involved a walk-around (Emmel & Clark, 2009) of areas of the home posing particular challenges to participants or where they had implemented assistive strategies.

For peer review only - http://bmjopen.bmj.com/site/about/guidelines.xhtml
For peer review only

Audio-recorded interview files were transcribed and a random portion checked for quality. All names and place names were changed.

Data analysis

Interview transcripts were uploaded into Atlas.ti qualitative data analysis software (version 7). The data were analysed using thematic analysis (Braun & Clarke, 2006) which was selected owing to its flexibility and accessibility. These were considered important factors given that this is the first qualitative exploration of individuals with PCA and also the wide range of health professionals who may find relevance in the findings. Two members of the research team (EH and MPS) were responsible for the analysis. They first familiarised themselves with the data with multiple read-throughs of transcripts before creating an initial coding framework based on existing literature on stress and coping in dementia, the study research questions and the initial familiarisation process. This coding framework was flexible and new codes added as required, following discussion and agreement. Each dyads' set of 3 interviews (person with PCA, family carer, joint) constituted one case for analysis and the cases were divided among the two authors EH and MPS. Once all 60 transcripts had been analysed and assigned initial codes, the codes were sorted into broader themes. Some codes were organised into major themes, some into sub themes and others became theme headings themselves. Themes and coded extracts within them were then reviewed in terms of their relevance, distinction from each other and coherence, and codes were reallocated or reorganised where required. Themes were then defined and named in such a way that they offered a coherent and consistent account of the data.

Quality assurance

Ongoing discussions acknowledge the complexity but necessity of assuring the quality of qualitative research. We draw on a notion of rigour in qualitative research suggested by Morse and colleagues (2002). Morse and colleagues (2002) identified five verification strategies for attaining reliability and validity in qualitative research which require consideration by the researchers throughout the research process, as opposed to criteria for reliability and validity which are determined post hoc and only by readers. Table 1 outlines these five verification strategies and where or how they are or were addressed in the design, conductance and write up of this study.

[Insert Table 1 here]

Member checking

For peer review only - http://bmjopen.bmj.com/site/about/guidelines.xhtml

Member checking is the process of presenting qualitative research findings to respondents or participants and inviting their feedback and/or checking for resonance of the findings with their own experiences (Morse et al., 2002). The advantages and disadvantages of member checking are much contested (e.g. Sandelowski, 1993; Harvey, 2015) and details of the debate are beyond the scope of this paper, but several issues bear particular relevance here. First, there is a concern that participants may inaccurately recall their original account and this is perhaps more likely in the case of progressive cognitive decline. Second, developments since the participants gave their original accounts may have changed their perceptions or how they may now choose to respond to the same questions, and this too may be of increased likelihood for those living with a progressive condition. For these potentially confounding reasons, we sought external validation of the current findings via two regional PCA support groups, made up of people with a diagnosis of PCA and their family carers (as in the study sample). Study findings were presented to both groups and comments invited, and there was a general consensus across both groups that the themes elicited here were compatible with support group members' own experiences. Many support group members went on to share their experiences by way of demonstrating the overlaps and coherence with the results of the current study.

To further establish the quality of the current research project, the project was conducted in accordance with the COREQ (COnsolidated criteria for REporting Qualitative research) criteria (Tong, Sainsbury & Craig, 2007) (see Appendix 1.)

Findings

Neuropsychological assessment

All participants with a diagnosis of PCA had previously (within 6 months) completed a selection of neuropsychological tests of memory, language and visual processing skills (visuoperceptual and visuospatial). Descriptive data relative to normative data sets appropriate for the mean age of the group are presented in Table 2. Mean participant scores on the Short Recognition Memory Test (Words), fragmented letters (visuoperceptual) and dot counting (visuospatial) tasks were below the 5th percentile, with mean scores on the concrete synonyms test falling within a normal range. About half of participants' individual scores on the tests of memory (Short Recognition Test for words) and language (concrete synonyms test) fell below the 5th percentile (n=11 and n=9 respectively), whereas almost all participants' individual scores on the tests of visuoperceptual (fragmented letters) and visuospatial (dot counting) processing skills fell below the 5th percentile (n=19 and n=18 respectively).

For peer review only - http://bmjopen.bmj.com/site/about/guidelines.xhtml

[Insert Table 2 here]

Qualitative interviews

Participants described a range of ways in which the diagnosis of PCA and the associated symptoms contributed to stress over time, and various strategies they and close others employed in response. The findings comprise three central themes that highlight some of the diagnosis-specific characteristics of PCA: the journey to diagnosis; interacting with the physical environment including managing activities of daily living, navigating the outside world, and use of aids and adaptations; and implications for the psychosocial environment such as maintaining independence and the adoption of a one day at a time approach to coping. Within these key themes there were associated temporal variations due to transitions associated with stage of life and others more representative of living with a neurodegenerative illness. The illness experience was framed by numerous contextual and background factors (e.g. existing relationship quality, personality factors [e.g. being a gregarious/ cautious/easy-going person], life stage transitions).

The journey to diagnosis

Initial symptoms were often described as incongruous or hard to pin-point, but nevertheless were an indication that something was wrong. Often, struggles with very familiar activities were first noticed:

Everything was hard for about a year, and [I was] beginning to feel there's something not right here, because I couldn't work out sort of basic things. (participant with PCA)

It was not uncommon for individuals to describe a problem that arose in their workplace, for example, reading financial accounts, or difficulties judging distance while driving. These challenges were often attributed to the health of their eyes and presented in stark contrast to the typical short-term memory problems first noticed in cases of tAD (e.g. Barnes et al., 2015).

With the everyday nature of tasks becoming difficult, this was inherently unsettling and a primary source of stress in and of itself. The majority (n=19) of individuals consulted eye-health professionals (e.g. optometrists) in the first instance, and underwent various inconclusive eye-health tests despite the cortical nature of their visual impairment. With hindsight, couples reflected on how the stress had been exacerbated and drawn out because of the lack of knowledge of the illness among the health-care professionals they were consulting. For some (n=10) this was complicated by concurrent eye-health issues, further delaying diagnosis. Experiences with general practitioners (n=17) were similarly reported to be

For peer review only - http://bmjopen.bmj.com/site/about/guidelines.xhtml

frustrating because of a lack of answers or appropriate and timely onwards referrals. For some, even a referral to a neurologist did not guarantee a diagnosis:

That would have saved me a lot of trouble if I'd believed myself. And it took ages, we went through about five or six neurologists /// Nothing. It was just dreadful because I kept thinking if I tell them what's wrong, what's happening and the symptoms, they're bound to know /// And nobody knew.

(participant with PCA)

Many families described stress caused by having to persist in their search for a diagnosis for what they considered to be an unacceptably long period of time. This is consistent with existing literature which describes the benefits of and need to prioritise the early diagnosis of dementia for individuals, families and society (e.g. Leifer, 2003; Robinson, Canavan & O'Keeffe, 2014), however this study highlights the particular barriers those with PCA face in receiving a timely diagnosis, owing to the rarity of the condition, associated lack of professional awareness and atypical symptom profile which lead them to exploring an eye-health route. Consistent with this idea of the importance of having knowledge of the illness, several participants explicitly stated the relief they experienced when the diagnosis was provided.

Following diagnosis, there was a widely reported lack of accessible information. This has previously also been reported by those adjusting to a diagnosis of tAD (e.g. Bunn et al. 2012; Clemerson, Walsh & Isaac, 2014) and young onset dementia specifically (e.g. Wawrziczny et al., 2016) and the current findings therefore add to knowledge about the varied types of dementia for which advice and information is needed. The rarity of PCA had the potential to be an ongoing source of stress over time in that those living with the diagnosis repeatedly found themselves better informed on the condition than health-care professionals they came into contact with, often having to re-explain the syndrome and their symptoms on multiple occasions.

Interacting with the physical environment

The nature of the symptoms (i.e. predominantly visual) meant effectively interacting with the physical environment was the predominant issue. This included interactions within and outside the home environment and with activities both functional and 'fun'.

Every participant described complications with completing self-care tasks, most prominently dressing and cooking. Difficulties with dressing included finding or selecting clothes or shoes, orienting them and using fastenings:

For peer review only - http://bmjopen.bmj.com/site/about/guidelines.xhtml

I do struggle a bit sometimes in working out which way round shirts go /// If it's all in a big heap, which it generally is, it's just a question of I will perhaps turn it round, sort of, two or three times before I work out where the collar is. (participant with PCA)

Dressing problems were exacerbated by distinguishing clean versus soiled clothes, seeing the closet and bedrooms being a shared space. Dressing assistance was frequently obtained from a family member or by decluttering, organising and simplifying the bedroom environment. In contrast to those with tAD, where problems with dressing may be attributable to problems with sequential task performance and attention (Namazi & Johnson, 1992), those with PCA articulately described clear visuo-percpetual and visuo-spatial processing problems underpinning their difficulties. Those with PCA were able to be similarly articulate about their choices and preferences around clothing and remained motivated to initiate dressing activities, which may be in contrast to those with tAD whose dressing may more commonly be disrupted by temporal disorientation and lack of motivation (e.g. Teri et al., 1989).

With cooking, typical problems were with locating ingredients/equipment in the kitchen, reading labels, following recipes, using appliances, or confidently and safely handling hot materials. For cooking or other household tasks, some individuals attempted to use visually salient strategies such as labelling cupboards or putting a red dot on the start button of an appliance. The effectiveness of these strategies were varied and more typically individuals retreated from these activities. As with dressing, when these difficulties are reported in the literature on tAD they are usually attributable to declining executive function skills (e.g. Perry & Hodges., 1999).

Of particular importance to participants were difficulties they had in engaging with a wide range of hobbies and interests including reading, DIY, sports and arts activities. The impact of this seemed heightened by the typically young age of onset which saw most participants approaching retirement age or having recently retired, and therefore allocating and looking forward to increasing time related to leisure activities:

I think we thought we'd be going out to theatre and travelling, and things more, whereas I'm planning in 2015 to make it the year I'm going to go to the matinees, try that. But, travelling has virtually stopped. We were going to take John's mother to the Christmas markets, thinking, well, with her help, I can probably get John on and off the train, but it... with her breaking her hip, that's another thing, another holiday went because of that, and so we thought, this is the time, this will be the time in our life that we need to travel, and this is the time in life, for one reason or another, we can't. /// Yes, I think we thought this would be the golden years.

For peer review only - http://bmjopen.bmj.com/site/about/guidelines.xhtml

(family carer)

The stress at having to retreat from or renegotiate hobbies is consistent with a study which looked at aspects which are important for quality of life but challenged by dementia (e.g. Thorgrimsen et al., 2003). However the barriers here were largely due to the specific visuo-spatial and visuo-perceptual deficits, which is in contrast to a study by Giebel and colleagues (2016) in which carers described those with tAD as having difficulties with the initiation rather than performance of such activities.

Beyond the home environment, all participants had difficulties navigating the external environment either on foot, by car or on public transport. Particular challenges were way- finding through crowds, reading signs or maps, general orientation, and using stairs/escalators:

I'm usually quite okay here but the minute I step outside the door it all goes mad. /// Life goes a bit strange, yes. /// even inside the village can be a bit strange but definitely when I go catching buses and dealing with... interacting with people, in general, no, it's not great, not great ./// I don't know what happens but it goes mad, yes. Not all the time and not every time but I'm much less comfortable and avoid, now, going unless I really need to go into town.

(participant with PCA)

Participants commonly stated they relied on routine responses to the environment and cues within it (e.g. using the same underground line or bus) or environmental cues (e.g. recognising a street by a particular shop or church), but naturally the external environment is not a stable one. In the case of (e.g.) a disruption in public transportation or a lorry blocking the view of an environmental cue, problems arose such as the person with PCA getting lost or disoriented. It is important to note this was not due to forgetfulness, distractibility or other executive function deficits as might be expected in cases of tAD (e.g. Chiu et al., 2004; Pai & Jacobs, 2004), but because of problems accurately perceiving visual information about the environment which would help individuals to work out where they were in relation to their target destination.

The difficulties that participants described in interacting with their physical environments are compatible with the sorts of neuropsychological deficits documented in people with PCA in the literature. Deficits like visual crowding, simultanagnosia, spatial navigational problems and apraxia (e.g. Mendez, Ghajarania & Perryman, 2002; Tang-Wai et al., 2004; Yong et al., 2014) corroborate with the issues people described with locating and manipulating objects, reading and dressing in this study.

As described above, the tendency towards simplification and familiarity meant withdrawing from certain activities. In addition though, almost all participants described ongoing uncertainty and

For peer review only - http://bmjopen.bmj.com/site/about/guidelines.xhtml

unpredictability associated with the disease profile, commenting that the difficulties were not reliably ever-present. This uncertainty was once again exacerbated by a reported lack of disease-specific provision and guidance. A minority of participants opted for off-the-shelf adaptations for those with eye-health problems, for example a symbol cane (n=3), or had been in touch with the Royal National Institute for the Blind regarding visual aids (n=5) but most took a self-initiated, largely trial and error approach owing to the unusual, unpredictable and continually changing nature of the symptoms. This process was rife with uncertainty regarding if things would work, why they might not, and over time, how long they would continue to work:

Camilla can still read, so if it's just one word it's okay /// but we've done different colour coding [on shampoo/conditioner bottles] and this sort of stuff but then she forgets which colour's which. So it's not, you know, it seems to be simple…but then there'll be some other obstacle along the way.

(family carer)

Allen, Cain and Meyer recently (2017) described a similar trial and error approach to environmental adaptations being employed by community-dwelling people with tAD and their carers, but these were more often triggered by – and designed to ameliorate – difficulties associated with dominant memory problems (e.g. using labels as reminders).

Acknowledging the temporal context, the participant above like many others referred to what his wife could 'still' do – appearing to demonstrate an anticipation of continuing decline over time. This is something commonly reported throughout the dementia literature in general (e.g. Roach et al., 2008; Harris & Keady, 2009). In the case of PCA though, this was coupled with a relative paucity of accessible knowledge or professional guidance as to how the particular course of disease would progress.

Implications within the psychosocial environment

Overall, there were broader psychosocial ramifications arising from these day- to- day difficulties such as maintaining independence and contemplating an uncertain short and long term future.

The symptoms themselves and resulting difficulties in interactions within the physical environment naturally impacted individuals' ability to perform daily activities independently:

I think that is the worst thing I can do nothing for myself so all the time you've got to ask somebody

(participant with PCA)

For peer review only - http://bmjopen.bmj.com/site/about/guidelines.xhtml

Psychosocial strategies used to mediate the stress associated with performance of these daily activities were largely the provision of physical assistance and reallocation of responsibilities within the dyad or family. Over time this often resulted in increasing feelings of dependence:

One of my big problems is frustration that I can't do things I want to do. I don't need to do them but because I've always been able to do them it really irks me to have to phone my son up and say, come and put this together for me. (participant with PCA)

This example illustrates a common phenomenon reported in the interviews – the way this perceived dependency resulted in changing roles and responsibilities within families. The extent to which this caused secondary strain for individuals seemed to be mediated by contextual factors including the age of onset, personality factors and previous relationship quality. With age of onset, individuals had often been at the peak of their careers and/or in a critical position within the family system (e.g. looking after both children and elderly parents). Such activities and the roles they represented were often defining in terms of individuals' senses of self and identity:

…he does all the ironing and everything now. And I say, no, that's my job. /// I'd always done it, you know. Jack was always working and I had the children and everything, and just did it. /// And I must admit, I sit here now and I think, I can't do anything. (participant with PCA)

The threat that dementia and the associated decline in functioning can pose for a person's sense of identity and independence is well documented throughout the existing literature, even when functional capacity is disrupted by memory problems (e.g. Clare et al., 2008; Harris & Keady, 2009) rather than cortical visual deficits as seen here.

Family carers (n=13) also described concerns they had about their competencies in taking on the new role of caregiver and reported the strain of uncertainties in knowing how and when to provide help. Many noted trying to strike a balance between getting things done, preventing their family member's distress or frustration, and encouraging or facilitating their ongoing independence.

Any stress and strain for participants who had a diagnosis of PCA regarding the impact on identity, role and independence were arguably emphasised by their relatively intact memory functions and abilities and inclination to reflect on and compare their previous experiences with their current situations. One way this caused stress was that the person with PCA could reflect on themselves and their declining abilities and in doing so most (n=15) were concerned that they were becoming a burden to their partner or wider family. However, on the other hand, these relatively intact capacities were also able to contribute to relieving stress for the majority of couples who took an interdependent or 'teamwork' (n=16) approach to managing the difficulties, via continued

For peer review only - http://bmjopen.bmj.com/site/about/guidelines.xhtml

collaboration and joint problem solving. This was evident in shared story-telling during the joint interviews and in the corresponding and complimentary accounts given in the individual interviews. Individuals with PCA (n=11) expressed extensive gratitude or feeling 'lucky' for their spouse/family carer while also normalising any symptom-specific dependency as one of many ways in which they and their family member worked together to manage life's challenges:

If you get married, sickness and in health, you have to keep to these things… I'm old-fashioned enough to think... you know, if it were me, he would look after me, I have no doubt. /// So, you know, what I think, and this is what I say to him, we're married, we're two parts of a whole, so in many ways it affects me, because then when you are supposed to be as one, as a whole…then you have to look after the other half of you, and, you know, by keeping one half healthy, helps the other half.

(family carer)

There was a suggestion that a relatively preserved insight and ability to plan in people with PCA promoted a continuation of closeness and collaboration between dyads, potentially contrasting with previous studies involving participants with young onset- but memory-led, dementias. Baikie (2002) reported a loss of joint decision making in marital relationships while separateness made up part of an overarching theme in a study by O'Shaughnessy, Lee and Lintern (2010) about the impact of dementia on the marital relationship. Similarly, Wright (1991) described how a lack of awareness contributed to discrepancies in the accounts of people with dementia and their spouses regarding their experiences of tension within the relationship and also an overall reduction in shared meanings made about the illness experience. However, there are also reports of couples living with memory-led dementias taking a continued team-work approach (e.g. Bunn et al., 2012) and sustained reciprocity in consideration of the others' needs (Merrick, Camic & O'Shaughnessy, 2016), both of which serve as a useful reminder of our need to interpret with caution. Two points seem salient here. First, the dementias progress in different ways for different people. And secondly, given the variation, states of separateness or connectedness, or team-work versus independence, do not apply discretely and exclusively to a group of people with one diagnosis and not to another. The participants with PCA interviewed here will likely progress to have more memory challenges over time, potentially poses additional challenges to shared meaning-making, and those in the early stages of more typical dementias are increasingly shown to be able to reflect reliably and accurately on their own experiences and abilities (e.g. Menne, Kinney & Morhardt, 2002; Merrick, Camic & O'Shaughnessy, 2016), something essential for joint problem solving and shared decision-making.

Another factor to consider is the sociocultural context within which research questions are framed and studies carried out. As the value put on quality of life surpasses that of quantity and as cures for

For peer review only - http://bmjopen.bmj.com/site/about/guidelines.xhtml

dementias remain elusive, attention has shifted to ideas of preserving personhood of those with dementia and more recently still, the couple-hood of dyads living with a diagnosis. Acknowledging the person and not just their disease has also meant a shift in focus from seeking to document deficits, loss and failings and attempts to capture the whole breadth of experience as attempted here – the positives, strengths, closeness and resilience (e.g. Hellstrom, Nolan & Lundh, 2007; Molyneaux et al., 2011).

One day at a time

Beyond diagnosis and in terms of longer term ongoing coping with the diagnosis, uncertainty and a lack of knowledge persisted, this time with regards to what to expect and how the disease would progress:

There's little point in thinking about the future because, in that sense, one would have to have a model of what the future may hold and therein lies part of the difficulty, that I can't map that future and make any choices. (participant with PCA)

This impacted how able many dyads felt to effectively plan for the future and many described taking a conscious decision to not think about it, given that there seemed to be little purpose or pleasure in doing so because of the incurable and progressive nature of the diagnosis:

Every now and again I get down, mainly because if I think too long about what the future holds then … it's counter-productive /// It's going to happen. There's nothing you can do about it. You know, it's like one of those things. It's nothing... it's all... you can give a problem a lot of thought if there's an answer; right, do we do this or do we do that? Right, think about it a long time, perhaps worry about it for a couple of days. Right, let's do that. With this, there isn't... What's Plan B? You haven't got a Plan B. And that's this situation. There isn't a Plan B.

(participant with PCA)

In shifting the focus away from an uncertain future, many described their approach as being centred around ideas of 'keeping going' and 'getting on with things', with efforts to maintain normality as far as possible:

When she [my wife] got her head around what she had, she said, there's nothing I can do about it, we've just got to get on with it. And we just carried on as normal. /// When it crops up, I deal with it, but 99% of the time, we just carry on as normal. //// I mean, obviously you gradually get worse and

For peer review only - http://bmjopen.bmj.com/site/about/guidelines.xhtml

worse, but, you know.

(family carer)

This family carer's comment clearly demonstrates the complexity and significance of temporality in describing the simultaneous day-by-day approach that can exist in combination with broader acknowledgement and anticipation of ongoing decline over the long term.

There were several reports of professionals endorsing or echoing this approach of living in the moment:

She [doctor] just looked at him and... put her hands on his legs and said, just live your life /// just go on and live your life, that's all you can do. (family carer)

This comment also seemed to address the inevitability of the progression of the disease in describing taking this approach as the only thing participants could do in the face of the diagnosis (i.e. in the absence of a cure). Perhaps it also hints at the lack of published guidance and knowledge about progression of the disease and what to expect which may have enabled or assisted longer term care planning and management. This is corroborated by a recent paper which identified the challenges families and practitioners face in finding tailored, disease-specific information and practical advice about PCA which is evidence –based (Wilson et al., 2016).

Taking a day-by-day approach was not only preferred but required due to the ever-changing nature of the symptom profile as the disease progressed. Individuals and families were continually responsive and attuned to the necessary ongoing adjustments and adaptations required by the continual change that is characteristic of the disease profile:

Yes, as I say, if I let my mind go there [the future], I will probably collapse in a heap, so I find it's best just to deal with things as they present, and just try and think one step ahead, and not too far, because, as I keep being told, every individual with the disease is different, and they can make no...they've got no crystal balls to see into the future, about exactly how it's going to pan for any...one person (family carer)

The sense of needing to balance the maintenance of normality in the face of diagnosis-related changes which require ongoing adjustment is also commonly reported throughout the qualitative

For peer review only - http://bmjopen.bmj.com/site/about/guidelines.xhtml

For peer review only

literature on dementia (e.g. Menne, Kinney & Morhardt, 2002; O'Shaughnessy, Lee & Lintern 2010; Molyneaux, Butchard, Simpson & Murray 2011; Bunn et al., 2012). A decision to focus on the 'here and now' is also widely reported, owing to the uncertainty or discomfort associated with thoughts about the future, in line with what was reported here (e.g. O'Shaughnessy, Lee & Lintern 2010; Bunn et al., 2012). These similarities with existing literature perhaps highlight the progressive nature which is common to all variations of AD and dementias more broadly.

5. Discussion

This study has illustrated some of the diagnosis-specific stressors associated with PCA and the various ways individuals and families attempt to mediate these. The characteristic visually-dominated symptom profile led to primary difficulties in interacting with the physical environment. These were situated within a complex psychosocial environment involving a range of roles and responsibilities requiring reallocation and various individuals' longstanding preferences regarding independence versus asking for, accepting and providing help. The rarity of the condition meant a lack of knowledge and accessible information about the symptoms, disease course and provision of support for those living with PCA and healthcare professionals all of which contributed to stress. The temporal context was of particular significance in shaping the stress process in terms of the time of life (e.g. employment/retirement status; position in the family); previous levels of performance and engagement in activities/interests; consideration of and concern about the future; and the time-limited efficacy of support strategies.

This study offers an original contribution in looking at the day-to-day impacts of progressive visual impairment related to dementia rather than the more typical memory loss (e.g. Steeman et al., 2006). Also, the inductive, qualitative methodology used here offers unique insights to complement the existing PCA literature which is largely laboratory-based and concentrated on specifying the cognitive profile and underlying pathology of the disease. This study has gleaned insights firmly grounded in participants' day-to-day experiences, within their home environments and recounted in their own words. This has allowed the full range of experience to be reported and documented. For example, families were able to share the problematic nature of symptoms and ongoing decline, but also their resilience in their collaborative and creative approaches to developing coping strategies and in continually adapting to the diagnosis-related changes.

Although this is the first qualitative study of PCA the findings here bear relevance to existing literature in several ways. The stress caused by the uncertainty and atypical nature of the diagnosis

For peer review only - http://bmjopen.bmj.com/site/about/guidelines.xhtml

and the impact of lacking disease-specific support and guidance echoes that which other authors have described in relation to other rare diseases (e.g. Wallenius, Möller & Berglund, 2009). The findings here may also bear relevance for those living with or seeking diagnoses of other rarer types of dementias, into which research is rightfully ever-increasing (e.g. behavioural variant frontotemporal dementia and the primary progressive aphasias; see Ritchie & Lovestone, 2002).

Overall, we need to remain critical of the questions we ask and sensitive to the individual differences in disease profile across and within diagnostic groups – especially in terms of what this means in terms of psychosocial impact for the individual and family. Perhaps the most significant contribution of this paper is in outlining the different mechanisms (e.g. visual versus memory problems) which can underpin difficulties with daily activities – even if the psychosocial ramifications of such difficulties are similar or overlapping. Understanding the ways in which the experiences of those with different diagnoses and at different stages of their disease overlap and diverge will be essential if we are to build a knowledge base in which all the complex stories of living with the dementias are told.

A strength of the study is that those with a diagnosis of PCA and a family member were interviewed together and separately, ensuring that multiple perspectives were represented in the data set. In this case, and in contrast to some existing literature on spousal couples living with tAD (e.g. Wright, 1991; Baikie, 2002; O'Shaughnessy, Lee & Lintern, 2010) the relatively well preserved insight of those with PCA resulted in largely congruent accounts of both parties in terms of levels of abilities and shared understandings of the illness experience. That interviews were conducted in the home environment enriched the data in permitting researchers an in-depth understanding of the everyday physical environment in which difficulties emerged and were responded to and often acted as a useful prompt for participants when discussing challenges and strategies. Working within a process model encouraged consideration of related underlying mechanisms, resulting stressors and responsive coping strategies. The study also makes a broader contribution in highlighting a potential limitation of the Stress Process Model in not taking account of the physical environment as a potential source or mediator of stress, despite suggestions that this may play a particularly significant role for people with PCA (Tang-Wai et al., 2014) and dementia in general (O'Malley & Croucher, 2005; Fleming & Purandare, 2010; Woodbridge et al., 2016). A possible limitation of the study is that the home-based nature of the interviews may have deterred dyads who were not managing well from taking part, and as such the findings may not capture the full range of coping responses to the stress process. The interviews took place at one time point, and the emergent importance of the temporal context may make this another limitation of the study.

For peer review only - http://bmjopen.bmj.com/site/about/guidelines.xhtml

In light of these findings, implications for clinicians centre around the need for increased knowledge and provision of information – particularly in a diagnostic context – which is particular to the challenges associated with dementia-related visual impairment and sensitive to the psychosocial ramifications of these difficulties. In addition, dominant difficulties interacting with the physical world may make those with PCA particularly suitable for psychosocial interventions targeted at the marital or family unit as a whole, owing to the relative cognitive strengths of those with PCA (e.g. insight and memory). Unanswered questions remain about how PCA progresses beyond the moderate stages and also how visual problems may affect people with more typical, memory-led forms of dementia, perhaps at a later stage when they are less easily communicated. Future research that looks at this both over time and taking account of the multiple perspectives inherent in any dementia journey would constitute valuable and original contributions to knowledge.

6. Conclusion

This study provides new insights into the stress process for individuals and families living with PCA, from the search for a diagnosis through to the daily challenges of living with dementia-related visual impairment. Increased availability and accessibility of information about PCA, its early symptoms and progression for both healthcare professionals and affected families would be beneficial in aiding timely diagnosis and minimising ongoing stress and uncertainty. Key considerations in the design of supportive interventions for those with PCA would be timeliness and sensitivity to the complexities of the surrounding psychosocial environment within which they must be adopted and adapted to over time.

7. Acknowledgements

We would like to thank all those who participated in this study. This work was supported by Economic and Social Research Council (ESRC)-National Institute for Health Research (NIHR) (UK) grant ES/L001810/1. Grant title: Seeing what they see: compensating for cortical visual dysfunction in Alzheimer's Disease. SC is also supported by the NIHR Queen Square Dementia Biomedical Research Unit and an Alzheimer's Research UK Senior Research Fellowship. RW is supported on a Brunel University London doctoral scholarship.

8. Conflict of Interest

None.

For peer review only - http://bmjopen.bmj.com/site/about/guidelines.xhtml

9. Data Statement

Data will be made available in accordance with funder guidelines after the completion of the project (March 2018).

10. Author Statement

EH contributions to study protocol development; data collection, analysis and interpretation; manuscript preparation (drafting and incorporation of comments/amendments).

MPS contributions to study protocol development; data collection, analysis and interpretation; provided comments on draft manuscripts and approved final manuscript.

RW contributions to study protocol development; data collection; provided comments on draft manuscript.

KY contributions to study protocol development; recruitment; data collection, analysis and interpretation; provided comments on draft manuscript.

AM contributions to study protocol development; data collection, analysis and interpretation.

MG contributions to study protocol development; provided comments on draft manuscript.

KG contributions to study protocol development; comments on draft manuscript.

SC contributions to study protocol development; provided comments on draft manuscripts and approved final manuscript.

11. References

Alzheimer's Society (2014) *Dementia UK: Update. Second edition.* Alzheimer's Society, London.

Alzheimer's Society. (2015). *What is young-onset dementia?* [Fact sheet]. Retrieved from https://www.alzheimers.org.uk/site/scripts/download_info.php?fileID=1766

Baikie, E. (2002). The impact of dementia on marital relationships. *Sexual and Relationship Therapy, 17*(3), 289-299.

Bakker, C., de Vugt, M. E., Vernooij-Dassen, M., van Vliet, D., Verhey, F. R., & Koopmans, R. T. (2010). Needs in early onset dementia: a qualitative case from the NeedYD study. *American journal of Alzheimer's disease and other dementias*, *25*(8), 634-640.

For peer review only

For peer review only - http://bmjopen.bmj.com/site/about/guidelines.xhtml

Barnes, J., Dickerson, B. C., Frost, C., Jiskoot, L. C., Wolk, D., & van der Flier, W. M. (2015). Alzheimer's disease first symptoms are age dependent: evidence from the NACC dataset. *Alzheimer's & Dementia, 11*(11), 1349-1357.

Braun, V., & Clarke, V. (2006). Using thematic analysis in psychology. *Qualitative research in psychology, 3*(2), 77-101.

Bunn, F., Goodman, C., Sworn, K., Rait, G., Brayne, C., Robinson, L., ... & Iliffe, S. (2012). Psychosocial factors that shape patient and carer experiences of dementia diagnosis and treatment: a systematic review of qualitative studies. *Public Library of Science Medicine, 9*(10), e1001331.

Cahill, S., Woods, B., Droes, R. M., Husband, H., Burnham, M., Urbas, S., ... & Vernooij-Dassen, M. (2008). *Early psychosocial interventions in dementia: evidence-based practice*. Jessica Kingsley Publishers.

Charles, R. F., & Hillis, A. E. (2005). Posterior cortical atrophy: clinical presentation and cognitive deficits compared to Alzheimer's disease. *Behavioural Neurology, 16*(1), 15-23.

Chiu, Y. C., Algase, D., Whall, A., Liang, J., Liu, H. C., Lin, K. N., & Wang, P. N. (2004). Getting lost: Directed attention and executive functions in early Alzheimer's disease patients. *Dementia and geriatric cognitive disorders, 17*(3), 174-180.

Clare, L., Rowlands, J., Bruce, E., Surr, C., & Downs, M. (2008). 'I don't do like I used to do': A grounded theory approach to conceptualising awareness in people with moderate to severe dementia living in long-term care. *Social Science & Medicine, 66*(11), 2366-2377.

Clemerson, G., Walsh, S., & Isaac, C. (2014). Towards living well with young onset dementia: An exploration of coping from the perspective of those diagnosed. *Dementia, 13*(4), 451-466.

Crutch, S. J., Lehmann, M., Schott, J. M., Rabinovici, G. D., Rossor, M. N., & Fox, N. C. (2012). Posterior cortical atrophy. *The Lancet Neurology*, *11*(2), 170-178.

Crutch, S. J., Schott, J. M., Rabinovici, G. D., Murray, M., Snowden, J. S., van der Flier, W. M., ... & Boeve, B. F. (2017). Consensus classification of posterior cortical atrophy. *Alzheimer's & Dementia* (in press).

Emmel, N., & Clark, A. (2009). The methods used in connected lives: Investigating networks, neighbourhoods and communities. NCRM Working Paper. National Centre for Research Methods.

For peer review only - http://bmjopen.bmj.com/site/about/guidelines.xhtml

Fleming, R., & Purandare, N. (2010). Long-term care for people with dementia: environmental design guidelines. *International Psychogeriatrics, 22*(7), 1084-1096.

Giebel, C. M., Challis, D. J., & Montaldi, D. (2016). A revised interview for deterioration in daily living activities in dementia reveals the relationship between social activities and well-being. *Dementia, 15*(5), 1068-1081.

Gilhooly, K. J., Gilhooly, M. L. M., Sullivan, M. P., McIntyre, A., Wilson, L., Harding, E., ... & Crutch, S. (2016). A meta-review of stress, coping and interventions in dementia and dementia caregiving. *BMC Geriatrics, 16*(1), 106.

Griffin, J., Oyebode, J. R., & Allen, J. (2016). Living with a diagnosis of behavioural-variant frontotemporal dementia: The person's experience. *Dementia, 15*(6), 1622-1642.

Harris, P. B., & Keady, J. (2009). Selfhood in younger onset dementia: transitions and testimonies. *Aging and Mental Health, 13*(3), 437-444.

Harvey, L. (2015). Beyond member-checking: A dialogic approach to the research interview. *International Journal of Research & Method in Education, 38*(1), 23-38.

Hellstrom, I., Nolan, M., & Lundh, U. (2007). Sustaining couplehood: Spouse' strategies for living positively with dementia. *Dementia, 6*, 383–409.

Johannessen, A., & Möller, A. (2013). Experiences of persons with early-onset dementia in everyday life: a qualitative study. *Dementia, 12*(4), 410-424.

Judge, K. S., Menne, H. L., & Whitlatch, C. J. (2010). Stress process model for individuals with dementia. *The Gerontologist, 50*(3), 294-302.

Kasl-Godley, J., & Gatz, M. (2000). Psychosocial interventions for individuals with dementia: an integration of theory, therapy, and a clinical understanding of dementia. *Clinical Psychology Review, 20*(6), 755-782.

Lehmann, M., Crutch, S. J., Ridgway, G. R., Ridha, B. H., Barnes, J., Warrington, E. K., ... & Fox, N. C. (2011). Cortical thickness and voxel-based morphometry in posterior cortical atrophy and typical Alzheimer's disease. *Neurobiology of aging, 32*(8), 1466-1476.

Leifer, B. P. (2003). Early diagnosis of Alzheimer's disease: clinical and economic benefits. *Journal of the American Geriatrics Society, 51*(5s2).

For peer review only - http://bmjopen.bmj.com/site/about/guidelines.xhtml

Mendez, M. F., Ghajarania, M., & Perryman, K. M. (2002). Posterior cortical atrophy: clinical characteristics and differences compared to Alzheimer's disease. *Dementia and Geriatric Cognitive Disorders, 14*(1), 33-40.

Menne, H. L., Kinney, J. M., & Morhardt, D. J. (2002). 'Trying to Continue to Do as Much as They Can Do' Theoretical insights regarding continuity and meaning making in the face of dementia. *Dementia, 1*(3), 367-382.

Merrick, K., Camic, P. M., & O'Shaughnessy, M. (2016). Couples constructing their experiences of dementia: A relational perspective. *Dementia, 15*(1), 34-50.

Molyneaux, V. L., Butchard, S., Simpson, J., & Murray, C. (2011). The co-construction of couplehood in dementia. *Dementia, 11*, 483–502

Morgan, D. L., Ataie, J., Carder, P., & Hoffman, K. (2013). Introducing dyadic interviews as a method for collecting qualitative data. *Qualitative Health Research, 23*(9), 1276-1284.

Morse, J. M., Barrett, M., Mayan, M., Olson, K., & Spiers, J. (2002). Verification strategies for establishing reliability and validity in qualitative research. *International journal of qualitative methods, 1*(2), 13-22.

Namazi, K. H., & Johnson, B. D. (1992). Dressing independently: A closet modification model for Alzheimer's disease patients. *American Journal of Alzheimer's Care and Related Disorders & Research, 7*(1), 22-28.

O'malley, L., & Croucher, K. (2005). Housing and dementia care–a scoping review of the literature. *Health & social care in the community, 13*(6), 570-577.

O'Shaughnessy, M., Lee, K., & Lintern, T. (2010). Changes in the couple relationship in dementia care: Spouse carers' experiences. *Dementia, 9*(2), 237-258.

Pai, M. C., & Jacobs, W. J. (2004). Topographical disorientation in community-residing patients with Alzheimer's disease. *International journal of Geriatric Psychiatry, 19*(3), 250-255.

Paxton, J. L., Peavy, G. M., Jenkins, C., Rice, V. A., Heindel, W. C., & Salmon, D. P. (2007). Deterioration of visual-perceptual organization ability in Alzheimer's disease. *Cortex, 43*(7), 967-975.

Pearlin, L. I., Mullan, J. T., Semple, S. J., & Skaff, M. M. (1990). Caregiving and the stress process: An overview of concepts and their measures. *The Gerontologist, 30*(5), 583-594.

For peer review only

Perry, R. J., & Hodges, J. R. (1999). Attention and executive deficits in Alzheimer's disease: A critical review. *Brain, 122*(3), 383-404.

Renner, J. A., Burns, J. M., Hou, C. E., McKeel, D. W., Storandt, M., & Morris, J. C. (2004). Progressive posterior cortical dysfunction A clinicopathologic series. *Neurology, 63*(7), 1175-1180.

Ritchie, K., & Lovestone, S. (2002). The dementias. *The Lancet, 360*(9347), 1759-1766.

Roach, P., Keady, J., Bee, P., & Hope, K. (2008). Subjective experiences of younger people with dementia and their families: implications for UK research, policy and practice. *Reviews in Clinical Gerontology, 18*(2), 165-174.

Robinson, S. M., Canavan, M., & O'Keeffe, S. T. (2014). Preferences of older people for early diagnosis and disclosure of Alzheimer's disease (AD) before and after considering potential risks and benefits. *Archives of Gerontology and Geriatrics, 59*(3), 607-612.

Rubin, H. J., & Rubin, I. S. (2011). *Qualitative interviewing: The art of hearing data*. Sage.

Sandelowski, M. (1993). Rigor or rigor mortis: the problem of rigor in qualitative research revisited. *Advances in nursing science, 16*(2), 1-8.

Shakespeare, T. J., Yong, K. X., Foxe, D., Hodges, J., & Crutch, S. J. (2015). Pronounced impairment of everyday skills and self-care in posterior cortical atrophy. *Journal of Alzheimer's Disease*, *43*(2), 381-384.

Steeman, E., Casterlé, D., Dierckx, B., Godderis, J., & Grypdonck, M. (2006). Living with early-stage dementia: A review of qualitative studies. *Journal of advanced nursing, 54*(6), 722-738.

Suárez-González, A., Crutch, S. J., Franco-Macías, E., & Gil-Néciga, E. (2016). Neuropsychiatric symptoms in Posterior cortical atrophy and Alzheimer disease. *Journal of Geriatric Psychiatry and Neurology*, 29(2), 65-71.

Tang-Wai, D. F., Graff-Radford, N. R., Boeve, B. F., Dickson, D. W., Parisi, J. E., Crook, R., ... & Petersen, R. C. (2004). Clinical, genetic, and neuropathologic characteristics of posterior cortical atrophy. *Neurology, 63*(7), 1168-1174.

Tang-Wai, D. F., Lake, A., & Graff-Radford, N. (2014). Posterior cortical atrophy. *Dementia: Comprehensive Principles and Practices.*

Taylor, B., & de Vocht, H. (2011). Interviewing separately or as couples? Considerations of authenticity of method. *Qualitative Health Research, 21*(11), 1576-1587.

For peer review only - http://bmjopen.bmj.com/site/about/guidelines.xhtml

Teri, L., Borson, S., Kiyak, H. A., & Yamagishi, M. (1989). Behavioral disturbance, cognitive dysfunction, and functional skill: Prevalence and relationship in Alzheimer's disease. *Journal of the American Geriatrics Society 37*(2), 109-116.

Thorgrimsen, L., Selwood, A., Spector, A., Royan, L., de Madariaga Lopez, M., Woods, R. T., & Orrell, M. (2003). Whose quality of life is it anyway?: The validity and reliability of the Quality of Life-Alzheimer's Disease (QoL-AD) scale. *Alzheimer Disease & Associated Disorders, 17*(4), 201-208.

Tong, A., Sainsbury, P., & Craig, J. (2007). Consolidated criteria for reporting qualitative research (COREQ): a 32-item checklist for interviews and focus groups. *International Journal for Quality in Health Care, 19*(6), 349-357.

Victoroff, J., Ross, G. W., Benson, D. F., Verity, M. A., & Vinters, H. V. (1994). Posterior cortical atrophy: neuropathologic correlations. *Archives of Neurology, 51*(3), 269-274.

Wallenius, E., Möller, K., & Berglund, B. (2009). Everyday impact of having a rare diagnosis. A questionnaire study. *Nordic Journal of Nursing Research, 29*(3), 13-17.

Warrington, E. K. (1996). *The Camden memory tests: Manual* (Vol. 1). Psychology Press.

Warrington, E. K., & James, M. (1991). *The visual object and space perception battery*. Bury St Edmunds: Thames Valley Test Company.

Warrington, E. K., McKenna, P., & Orpwood, L. E. (1998). Single word comprehension: a concrete and abstract word synonym test. *Neuropsychological Rehabilitation, 8*(2), 143-154.

Wawrziczny, E., Pasquier, F., Ducharme, F., Kergoat, M. J., & Antoine, P. (2016). From 'needing to know'to 'needing not to know more': an interpretative phenomenological analysis of couples' experiences with early-onset Alzheimer's disease. *Scandinavian Journal of Caring Sciences, 30*(4), 695-703.

Wilson, L., Gilhooly, M. L. M., Sullivan, M. P., Gilhooly, K. J., McIntyre, A., Woodbridge, R., Harding, E., Walton, J., & Crutch, S. (2016). Posterior cortical atrophy: resources and information. *Journal of Dementia Care, 24*(1), 32-34.

Woodbridge, R., Sullivan, M. P., Harding, E., Crutch, S., Gilhooly, K. J., Gilhooly, M. L. M., ... & Wilson, L. (2016). Use of the physical environment to support everyday activities for people with dementia: A systematic review. *Dementia*, 1471301216648670.

For peer review only - http://bmjopen.bmj.com/site/about/guidelines.xhtml

Wright, L. K. (1991). The impact of Alzheimer's disease on the marital relationship. *The Gerontologist, 31*(2), 224-237.

Yong, K. X., Shakespeare, T. J., Cash, D., Henley, S. M., Warren, J. D., & Crutch, S. J. (2014). (Con) text-specific effects of visual dysfunction on reading in posterior cortical atrophy. *Cortex, 57*, 92-106.

Yong, K. X., Shakespeare, T. J., Cash, D., Henley, S. M., Nicholas, J. M., Ridgway, G. R., ... & Schott, J. M. (2014). Prominent effects and neural correlates of visual crowding in a neurodegenerative disease population. *Brain, 137*(12), 3284-3299.

For peer review only

For peer review only - http://bmjopen.bmj.com/site/about/guidelines.xhtml

Table 1. *Five verification strategies for attaining reliability and validity in qualitative research (Morse et al. 2002)*

Verification strategy	Explanation	How or where demonstrated in the current study
Methodological coherence	Ensuring congruence between research question and methods	Background (Stress Process Model)<br>Methods (design/sampling i.e. community-based sample; data collection i.e. individual and dyadic interviews in order to gain both shared and individual perspectives; data analysis, i.e. qualitative approach for study of little-known topic)
Appropriate sampling	Participants who best represent or have knowledge of the research topic	Methods (design/sampling i.e. community-based sample; data collection i.e. individual and dyadic interviews in order to gain both shared and individual perspectives; broad range of disease severity)
Collecting and analysing data concurrently	Establishing an iterative interaction between what is known and what one needs to know	Methods – moving between data collection and data analysis, including:<br>Memo-writing (keeping an ongoing log of analytical thoughts and ideas)<br>Amendments to interview schedule (adding questions/prompts to further explore emerging areas of interest e.g. role changes)<br>Field notes (written together by the two interviewing authors – MPS & RW or RW & EH – immediately after the interview to document initial responses and reflections on the data collected)
Thinking theoretically	Constant, cyclical process of checking that emerging ideas are reconfirmed in new data	Methods – moving between data collection and data analysis, including:<br>Memo-writing (keeping an ongoing log of analytical thoughts and ideas)<br>Amendments to interview schedule (adding

For peer review only

		questions/prompts to further explore emerging areas of interest e.g. role changes)<br>Field notes (written together by the two interviewing authors – MPS & RW or RW & EH – immediately after the interview to document initial responses and reflections on the data collected)
Theory development	Moving between micro-perspective to macro conceptual/theoretical understanding	Results (Major and sub themes, supporting quotes and explanatory commentary)<br>Discussion (compatibility with existing literature e.g. empirical – relationship impact and theoretical – utility of the Stress Process Model; research and clinical practice implications; suggestions for future work e.g. other rare dementia populations)

For peer review only

Table 2. *Neuropsychological scores of patients with PCA (N=20)*

Test	Max. score	Raw score (mean; SD *range*)	N < 5th %ile cut off	Comments
Short Recognition Memory Test[a] for words[b] [Memory]	25	17.98 ± 4.857 *12-25*	10	~ < 5th %ile (cut off 19)
Concrete Synonyms test[c] [Language]	25	21.33 ± 3.105 *12-25*	7	25th-50th %ile (cut off 18)
Fragmented letters (VOSP)[d] [Visuoperceptual]	20	1.60 ± 2.703 *0-10*	19	~ < 5th %ile (cut off 16)
Dot counting (VOSP)[d] [Visuospatial]	10	2.15 ± 2.961 *0-11*	18	~ < 5th %ile (cut off 8)

[a.] Behavioural screening tests supportive of PCA diagnosis.

[b.] Warrington (1996).

[c.] Warrington, McKenna and Orpwood (1998).

[d.] Visual Object and Space Perception Battery (VOSP; Warrington & James, 1991).

For peer review only

COREQ (COnsolidated criteria for REporting Qualitative research) Checklist

A checklist of items that should be included in reports of qualitative research. You must report the page number in your manuscript where you consider each of the items listed in this checklist. If you have not included this information, either revise your manuscript accordingly before submitting or note N/A.

Topic	Item No.	Guide Questions/Description	Reported on Page No.
**Domain 1: Research team and reflexivity**			
Personal characteristics			
Interviewer/facilitator	1	Which author/s conducted the interview or focus group?	7
Credentials	2	What were the researcher's credentials? E.g. PhD, MD	1
Occupation	3	What was their occupation at the time of the study?	-
Gender	4	Was the researcher male or female?	1
Experience and training	5	What experience or training did the researcher have?	1
Relationship with participants			
Relationship established	6	Was a relationship established prior to study commencement?	4
Participant knowledge of the interviewer	7	What did the participants know about the researcher? e.g. personal goals, reasons for doing the research	4-5
Interviewer characteristics	8	What characteristics were reported about the inter viewer/facilitator? e.g. Bias, assumptions, reasons and interests in the research topic	-
**Domain 2: Study design**			
Theoretical framework			
Methodological orientation and Theory	9	What methodological orientation was stated to underpin the study? e.g. grounded theory, discourse analysis, ethnography, phenomenology, content analysis	4-6
Participant selection			
Sampling	10	How were participants selected? e.g. purposive, convenience, consecutive, snowball	4-5
Method of approach	11	How were participants approached? e.g. face-to-face, telephone, mail, email	-
Sample size	12	How many participants were in the study?	1, 4-5
Non-participation	13	How many people refused to participate or dropped out? Reasons?	4-5
Setting			
Setting of data collection	14	Where was the data collected? e.g. home, clinic, workplace	1,5
Presence of non-participants	15	Was anyone else present besides the participants and researchers?	4-5
Description of sample	16	What are the important characteristics of the sample? e.g. demographic data, date	4-5
Data collection			
Interview guide	17	Were questions, prompts, guides provided by the authors? Was it pilot tested?	5-7
Repeat interviews	18	Were repeat inter views carried out? If yes, how many?	-
Audio/visual recording	19	Did the research use audio or visual recording to collect the data?	5
Field notes	20	Were field notes made during and/or after the inter view or focus group?	7
Duration	21	What was the duration of the inter views or focus group?	5
Data saturation	22	Was data saturation discussed?	-
Transcripts returned	23	Were transcripts returned to participants for comment and/or	5, 7

For peer review only

Topic	Item No.	Guide Questions/Description	Reported on Page No.
		correction?	
**Domain 3: analysis and findings**			
Data analysis			
Number of data coders	24	How many data coders coded the data?	5-6
Description of the coding tree	25	Did authors provide a description of the coding tree?	-
Derivation of themes	26	Were themes identified in advance or derived from the data?	5-7
Software	27	What software, if applicable, was used to manage the data?	5
Participant checking	28	Did participants provide feedback on the findings?	8
Reporting			
Quotations presented	29	Were participant quotations presented to illustrate the themes/findings? Was each quotation identified? e.g. participant number	10-18
Data and findings consistent	30	Was there consistency between the data presented and the findings?	10-18
Clarity of major themes	31	Were major themes clearly presented in the findings?	10-18
Clarity of minor themes	32	Is there a description of diverse cases or discussion of minor themes?	10-18

Developed from: Tong A, Sainsbury P, Craig J. Consolidated criteria for reporting qualitative research (COREQ): a 32-item checklist for interviews and focus groups. *International Journal for Quality in Health Care*. 2007. Volume 19, Number 6: pp. 349 – 357

Once you have completed this checklist, please save a copy and upload it as part of your submission. DO NOT include this checklist as part of the main manuscript document. It must be uploaded as a separate file.

For peer review only

BMJ Open

‘Because my brain isn’t as active as it should be, my eyes don’t always see’ – a qualitative exploration of the stress process for those living with posterior cortical atrophy

Journal:	*BMJ Open*
Manuscript ID	bmjopen-2017-018663.R1
Article Type:	Research
Date Submitted by the Author:	08-Nov-2017
Complete List of Authors:	Harding, Emma; University College London Institute of Neurology, Dementia Research Centre<br>Sullivan, Mary Pat; Nipissing University, School of Human and Social Development; University College London Institute of Neurology, Dementia Research Centre<br>Woodbridge, Rachel; Brunel University, Department of Clinical Sciences<br>Yong, Keir; University College London Institute of Neurology, Dementia Research Centre<br>McIntyre, Anne; Brunel University, Department of Clinical Sciences<br>Gilhooly, Mary; Brunel University, School of Health Science and Social Care<br>Gilhooly, KJ; Brunel University, Department of Clinical Sciences<br>Crutch, Sebastian; University College London Institute of Neurology
**Primary Subject Heading**:	Qualitative research
Secondary Subject Heading:	Neurology
Keywords:	Dementia < NEUROLOGY, EDUCATION & TRAINING (see Medical Education & Training), Neuro-ophthalmology < NEUROLOGY, PRIMARY CARE, QUALITATIVE RESEARCH

SCHOLARONE™
Manuscripts

'Because my brain isn't as active as it should be, my eyes don't always see' – a qualitative exploration of the stress process for those living with posterior cortical atrophy

Word count: 6943

Keywords: [MeSH terms] Alzheimer Disease, Dementia, Vision Disorders, Family, Qualitative Research

Authors and affiliations

Emma Harding[1] BSc., Mary Pat Sullivan[2] PhD., Rachel Woodbridge[3] BSc., Keir Yong[1] PhD., Anne McIntyre[3] PhD., Mary L. Gilhooly[3] PhD., Kenneth J. Gilhooly[3] PhD., Sebastian J. Crutch[1] PhD.

[1.] Dementia Research Centre, University College London, London, UK

[2.] Faculty of Applied and Professional Studies, Nipissing University, North Bay, Canada

[3.] Department of Clinical Sciences, Brunel University London, Uxbridge, UK

Correspondence to:

Emma Harding

Box 16, National Hospital for Neurology and Neurosurgery

Queen Square

London, WC1N 3BG

emma.harding@ucl.ac.uk

020 3488 3667 (Mondays and Fridays)

1. Abstract

Objectives: To explore the stress process for individuals living with posterior cortical atrophy (PCA) and their families.

Design: A qualitative study using in-depth semi-structured dyadic and individual interviews with people living with a diagnosis of PCA and a family carer. Interview transcripts were thematically analysed.

Setting: Participants' homes.

For peer review only

Participants: 20 individuals in the mild to moderate stages of PCA and 20 family carers.

Findings: Three major themes were identified – (1) The diagnostic journey: mostly an unsettling and convoluted process, owing to the early age of onset, rarity and atypical symptom profile of PCA. (2) Interactions with the physical environment: profound difficulties with functional and leisure activities were usually compensated for with adaptations maximising familiarity or simplicity. (3) Implications within the psychosocial environment: symptoms impacted individuals' sense of independence and identity and required reallocations of roles and responsibilities. Ongoing uncertainties and the progressive nature of PCA caused most dyads to take a 'one day at a time' approach to coping. Relatively well-preserved insight and memory were a benefit and burden, as individuals shared the illness experience with family members but also compared their current situation to before diagnosis. The experience was framed by background and contextual factors and understood within an ever-changing temporal context.

Conclusion: The stress process in PCA is characterised by uncertainty and unpredictability from diagnosis through to ongoing management. The provision of tailored information about cortical visual problems and associated functional difficulties, time-sensitive environmental adaptations to help those with PCA to identify what and where things are, and psychosocial interventions for the marital/family unit as a whole would be useful to improve both functional status and psychological wellbeing. Future research exploring (i) stress and coping in the later stages of PCA and (ii) the nature and impact of visual impairment(s) in typical Alzheimer's disease would be worthwhile.

2. Strengths and limitations of this study

- As the first qualitative study of those living with PCA this paper provides original, in-depth insights into the subjective experiences of those with dementia-related visual impairment.
- As well as providing empirical description of the illness experience, in using a conceptual framework (the Stress Process Model) this study also makes a broader contribution to social science and the field of dementia research.
- With both individual and dyadic interviews, this study allowed the multiple perspectives of both people with the diagnosis and their family carer – individual and shared narratives – to emerge.
- Conducting interviews in participants' homes permitted a rich understanding of the physical and psychosocial context within which daily difficulties owing to visual processing problems emerged.

For peer review only - http://bmjopen.bmj.com/site/about/guidelines.xhtml

- Interviews were conducted at one time point but owing to the progressive nature of the disease, future longitudinal research would be valuable to develop the current study findings.
- The interview method relies on participants' abilities to accurately recall their own experiences and while those with PCA, especially in the earlier stages, can have relatively well preserved memory, disease severity varied across the current sample and a sub-group of those interviewed were demonstrating some memory impairment during interview.

3. Background

There are an estimated 850,000 people currently living with dementia in the UK[1] and it is estimated that 5% of these (approx. 42,500) are cases of young onset dementias, with symptoms beginning before the age of 65[2]. Posterior Cortical Atrophy, originally called Benson's disease, is a rare form of dementia which is typically early in onset with symptoms usually beginning between the ages of 50 and 65 years[3]. The underlying pathology in the majority of individuals is Alzheimer's disease (AD) although a small number of cases attributable to Lewy Body disease and corticobasal degeneration have been reported[4 5]. The prevalence is unknown. PCA is characterised by initial neurodegeneration towards the back of the brain, specifically in the parietal, occipital, and occipitotemporal regions[6]. Correspondingly, the initial symptoms predominantly relate to cortical visual impairment, particularly deficits in visuo-spatial and visuo-perceptual processing. Other characteristic symptoms relate to impairments associated with posterior functions, including literacy, spelling and numeracy[6-7]. While current clinical criteria cite visual processing impairments with proportionally less impaired memory as core diagnostic features of PCA , patients may in fact exhibit memory impairments at initial presentation[4 8 9]. When compared to other dementias PCA is relatively under-researched. The majority of research into PCA is concentrated around establishing the neuropsychological, cognitive and imaging profile(s) of those with the diagnosis[4 9-11]. Anecdotal and laboratory based evidence suggests ways in which PCA might impact on people's daily lives, including problems with reading, driving and localising objects in space[9 12-14], but there is a paucity of research focusing on the everyday impact of living with the diagnosis for individuals and families.

One recent paper investigated the impact of PCA on activities of daily living, documenting difficulties with performance of everyday skills for people with PCA (including operation of appliances, writing and handling money) and self-care (including dressing, feeding and bathing) compared to

For peer review only - http://bmjopen.bmj.com/site/about/guidelines.xhtml

predominant deficits in memory, motivation and orientation of people with typical, amnestic presentations of Alzheimer's disease (tAD)[15]. In another recent paper, Suárez-González et al.[16] investigated the neuropsychiatric profile of people with PCA. They found similar increases in depression, apathy, irritability and anxiety for those with PCA to participants with tAD.

In the absence of disease modifying therapies for Alzheimer's disease and other forms of dementia, environmental and psychosocial interventions to improve the quality of life and wellbeing of those living with it hold particular significance[17-19]. Better understanding the needs of those with different rare and/or young onset dementias will be an important step in developing effective environmental or psychosocial interventions. A second generation of literature is beginning to delineate the particular experiences of those with less common forms of dementias such as young onset dementias[20-21] and those with atypical symptom profiles like behavioural variant frontotemporal dementia[22]. Developing a greater understanding of the day-to-day impact of dementia in relation to visual problems will be a timely addition to this, not least because those with typical amnestic presentations of AD may also go on to have cortical visual impairment, likely later on in their diagnosis and as such at a time when they may not be so able to articulate their experiences of the symptoms[23].

These varied everyday impacts of dementias which pose challenges for quality of life and wellbeing are often considered within a broad conceptual category of stressors, defined as demands which are considered to exceed a person's available resources. Cognitive, emotional and/or behavioural attempts to manage these demands are often approached and studied as coping strategies. Much attention has consistently been given over the past few decades to understanding what contributes to informal caregiver stress and what facilitates coping, given the huge societal contribution informal carers make by continuing to care for loved ones with dementia at home[24-25]. The stress-coping approach is widely acknowledged as dominant within this literature[26-27] and its popularity is exemplified by the numerous reviews into stress and coping in dementia noted by Gilhooly et al.[19]. There are multitudes of studies looking at the chronic and particular psychological, socioemotional and practical stressors and strains faced by carers of people with dementia[28-29] and also the nature and efficacy of the many practical, emotional, psychological and social coping strategies employed to mediate this[30-31].

The current study sought to maximise on the relative abilities of those with PCA to reflect on and communicate their experiences. Using the Stress Process Model[32-33] as a conceptual framework we present findings from a qualitative exploration of the stresses associated with mild to moderate stage PCA and responses to these over time. The Stress Process Model outlines primary stressors

For peer review only - http://bmjopen.bmj.com/site/about/guidelines.xhtml

which result directly from the disease itself, secondary strains which may follow, and both internal and external factors which mediate both of these in shaping outcomes. Having been developed to conceptualise informal caregivers' experiences[32], it has since been adapted for individuals with dementia[33], and in doing so acknowledges the multiple perspectives to be taken into account when understanding the dementia experience. More specifically, the study aimed to explore the potential of the physical environment to contribute to and/or mediate the stress process owing to the prominent visuo-spatial and visuo-perceptual symptoms.

4. Methods

Design/sampling

A qualitative design was deemed appropriate in order to gain rich detailed accounts and for possibly unanticipated insights to emerge[34]. This was considered important because of both the paucity of knowledge about the impact of PCA on individuals' subjective experiences and also the distinctiveness of the symptom profile. In-depth semi-structured interviews were chosen owing to the abilities of those with PCA to recount their experiences and again, to facilitate the collection of data of sufficient richness and depth. Participants were recruited via the Specialist Cognitive Disorders Clinic at the National Hospital for Neurology and Neurosurgery, University College London Hospitals NHS Foundation Trust. Inclusion criteria were a confirmed diagnosis of PCA and an accompanying family member or familiar other also willing to participate.

Twenty individuals with PCA (12 F; 8 M) took part in the interviews and the mean age was 68 years (7.66 SD). Scores on the Mini Mental State Examination (MMSE) ranged from 10-29 (mean=20.05; SD=6.54), indicating mild to moderate dementia. Twenty spouses/family carers took part (10 F; 10 M); in 18 cases this was a spouse, in one case the dyadic relationship was mother-daughter and in the other case it was aunt-niece. One spouse (F) opted not to take part in an individual interview, but did participate in the dyadic one. Only one participant lived alone. The number of years since diagnosis ranged from 0 to 12 (mean=3.31; SD=2.75) and the number of years since subjective onset ranged from 2 to 14 (mean=6.39; SD=3.26).

A comparative sample of people living with typical Alzheimer's disease (n=17) and their family carers (n=17) were also interviewed and findings from the subsequent analysis of that data will be reported in another paper.

Ethical approval

For peer review only - http://bmjopen.bmj.com/site/about/guidelines.xhtml

The study was approved by the National Research Ethics Service Committee – London Queen Square and informed consent was obtained from all participants. After the interviews the researchers conducted a short debrief providing further information and contact details in case of any issues or causes for concern related to the study.

Data collection

Individual and dyadic interviews were conducted at participants' homes (by EH & RW or MPS & RW). Dyads were interviewed together and then separately in order to capture the dyads' shared experience[35], allow the family carer to supplement the person with PCA's account in the case of additional, secondary memory impairment and to provide the opportunity for individuals to provide information they might not feel comfortable to disclose in the presence of their family member[36]. The interview schedule covered contextual factors (personal, marital and occupational history, current family situation), the diagnostic journey, and daily difficulties and coping strategies within the home environment. In total interviews lasted between 3-4 hours per dyad. The home visit also involved a walk-around[37] of areas of the home posing particular challenges to participants or where they had implemented assistive strategies.

Audio-recorded interview files were transcribed and a random portion checked for quality. All names and place names were changed.

Data analysis

Interview transcripts were uploaded into Atlas.ti qualitative data analysis software (version 7). The data were analysed using thematic analysis[38] which was selected owing to its flexibility and accessibility. These were considered important factors given that this is the first qualitative exploration of individuals with PCA and also the wide range of health professionals who may find relevance in the findings. Two members of the research team (EH and MPS) were responsible for the analysis. They first familiarised themselves with the data with multiple read-throughs of transcripts before creating an initial coding framework based on existing literature on stress and coping in dementia, the study research questions and the initial familiarisation process. This coding framework was flexible and new codes added as required, following discussion and agreement. Each dyads' set of 3 interviews (person with PCA, family carer, joint) constituted one case for analysis and the cases were divided among the two authors EH and MPS. Once all 60 transcripts had been analysed and assigned initial codes, the codes were sorted into broader themes. Some codes were organised into major themes, some into sub themes and others became theme headings themselves. Themes and coded extracts within them were then reviewed in terms of their relevance, distinction from each

For peer review only - http://bmjopen.bmj.com/site/about/guidelines.xhtml

other and coherence, and codes were reallocated or reorganised where required. Themes were then defined and named in such a way that they offered a coherent and consistent account of the data.

Quality assurance

Ongoing discussions acknowledge the complexity but necessity of assuring the quality of qualitative research. We draw on a notion of rigour in qualitative research suggested by Morse et al.[39]. Morse et al.[39] identified five verification strategies for attaining reliability and validity in qualitative research which require consideration by the researchers throughout the research process, as opposed to criteria for reliability and validity which are determined post hoc and only by readers. Table 1 outlines these five verification strategies and where or how they are or were addressed in the design, conductance and write up of this study.

Table 1. *Five verification strategies for attaining reliability and validity in qualitative research*[39]

**Verification strategy**	**Explanation**	**How or where demonstrated in the current study**
Methodological coherence	Ensuring congruence between research question and methods	Background (Stress Process Model)<br>Methods (design/sampling i.e. community-based sample; data collection i.e. individual and dyadic interviews in order to gain both shared and individual perspectives; data analysis, i.e. qualitative approach for study of little-known topic)
Appropriate sampling	Participants who best represent or have knowledge of the research topic	Methods (design/sampling i.e. community-based sample; data collection i.e. individual and dyadic interviews in order to gain both shared and individual perspectives; broad range of disease severity)
Collecting and analysing data concurrently	Establishing an iterative interaction between what is known and what one needs to know	Methods – moving between data collection and data analysis, including:<br>Memo-writing (keeping an ongoing log of analytical thoughts and ideas)<br>Amendments to interview schedule (adding questions/prompts to further explore emerging areas of interest e.g. role

For peer review only - http://bmjopen.bmj.com/site/about/guidelines.xhtml

		changes)<br>Field notes (written together by the two interviewing authors – MPS & RW or RW & EH – immediately after the interview to document initial responses and reflections on the data collected)
Thinking theoretically	Constant, cyclical process of checking that emerging ideas are reconfirmed in new data	Methods – moving between data collection and data analysis, including:<br>Memo-writing (keeping an ongoing log of analytical thoughts and ideas)<br>Amendments to interview schedule (adding questions/prompts to further explore emerging areas of interest e.g. role changes)<br>Field notes (written together by the two interviewing authors – MPS & RW or RW & EH – immediately after the interview to document initial responses and reflections on the data collected)
Theory development	Moving between micro-perspective to macro conceptual/theoretical understanding	Results (Major and sub themes, supporting quotes and explanatory commentary)<br>Discussion (compatibility with existing literature e.g. empirical – relationship impact and theoretical – utility of the Stress Process Model; research and clinical practice implications; suggestions for future work e.g. other rare dementia populations)

Member checking

Member checking is the process of presenting qualitative research findings to respondents or participants and inviting their feedback and/or checking for resonance of the findings with their own experiences[39]. The advantages and disadvantages of member checking are much contested[40-41] and details of the debate are beyond the scope of this paper, but several issues bear particular relevance

For peer review only - http://bmjopen.bmj.com/site/about/guidelines.xhtml

here. First, there is a concern that participants may inaccurately recall their original account and this is perhaps more likely in the case of progressive cognitive decline. Second, developments since the participants gave their original accounts may have changed their perceptions or how they may now choose to respond to the same questions, and this too may be of increased likelihood for those living with a progressive condition. For these potentially confounding reasons, we sought external validation of the current findings via two regional PCA support groups, made up of people with a diagnosis of PCA and their family carers (as in the study sample). Study findings were presented to both groups and comments invited, and there was a general consensus across both groups that the themes elicited here were compatible with support group members' own experiences. Many support group members went on to share their experiences by way of demonstrating the overlaps and coherence with the results of the current study.

To further establish the quality of the current research project, the project was conducted in accordance with the COREQ (COnsolidated criteria for REporting Qualitative research) criteria[42] (see Appendix 1.)

Findings

Neuropsychological assessment

All participants with a diagnosis of PCA had previously (within 6 months) completed a selection of neuropsychological tests of memory, language and visual processing skills (visuoperceptual and visuospatial). Descriptive data relative to normative data sets appropriate for the mean age of the group are presented in Table 2. Mean participant scores on the Short Recognition Memory Test (Words), fragmented letters (visuoperceptual) and dot counting (visuospatial) tasks were below the 5th percentile, with mean scores on the concrete synonyms test falling within a normal range. About half of participants' individual scores on the tests of memory (Short Recognition Test for words) and language (concrete synonyms test) fell below the 5th percentile (n=11 and n=9 respectively), whereas almost all participants' individual scores on the tests of visuoperceptual (fragmented letters) and visuospatial (dot counting) processing skills fell below the 5th percentile (n=19 and n=18 respectively).

For peer review only - http://bmjopen.bmj.com/site/about/guidelines.xhtml

Table 2. *Neuropsychological scores of patients with PCA (N=20)*

Test	Max. score	Raw score (mean; SD *range*)	N < 5th %ile cut off	Comments
Short Recognition Memory Test[a] for words[b] [Memory]	25	17.98 ± 4.857 *12-25*	10	~ < 5th %ile (cut off 19)
Concrete Synonyms test[c] [Language]	25	21.33 ± 3.105 *12-25*	7	25th-50th %ile (cut off 18)
Fragmented letters (VOSP)[d] [Visuoperceptual]	20	1.60 ± 2.703 *0-10*	19	~ < 5th %ile (cut off 16)
Dot counting (VOSP)[d] [Visuospatial]	10	2.15 ± 2.961 *0-11*	18	~ < 5th %ile (cut off 8)

a. Behavioural screening tests supportive of PCA diagnosis.

b. [43]

c. [44]

d. [45]

Qualitative interviews

Participants described a range of ways in which the diagnosis of PCA and the associated symptoms contributed to stress over time, and various strategies they and close others employed in response. The findings comprise three central themes that highlight some of the diagnosis-specific characteristics of PCA: the journey to diagnosis; interacting with the physical environment including managing activities of daily living, navigating the outside world, and use of aids and adaptations; and implications for the psychosocial environment such as maintaining independence and the adoption of a one day at a time approach to coping. Within these key themes there were associated temporal variations due to transitions associated with stage of life and others more representative of living with a neurodegenerative illness. The illness experience was framed by numerous contextual and background factors (e.g. existing relationship quality, personality factors [e.g. being a gregarious/ cautious/easy-going person], life stage transitions).

The journey to diagnosis

For peer review only - http://bmjopen.bmj.com/site/about/guidelines.xhtml

Initial symptoms were often described as incongruous or hard to pin-point, but nevertheless were an indication that something was wrong. Often, struggles with very familiar activities were first noticed:

Everything was hard for about a year, and [I was] beginning to feel there's something not right here, because I couldn't work out sort of basic things. (participant with PCA)

It was not uncommon for individuals to describe a problem that arose in their workplace, for example, reading financial accounts, or difficulties judging distance while driving. These challenges were often attributed to the health of their eyes and presented in stark contrast to the typical short-term memory problems first noticed in cases of tAD[46].

With the everyday nature of tasks becoming difficult, this was inherently unsettling and a primary source of stress in and of itself. The majority (n=19) of individuals consulted eye-health professionals (e.g. optometrists) in the first instance, and underwent various inconclusive eye-health tests despite the cortical nature of their visual impairment. With hindsight, couples reflected on how the stress had been exacerbated and drawn out because of the lack of knowledge of the illness among the health-care professionals they were consulting. For some (n=10) this was complicated by concurrent eye-health issues, further delaying diagnosis. Experiences with general practitioners (n=17) were similarly reported to be frustrating because of a lack of answers or appropriate and timely onwards referrals. For some, even a referral to a neurologist did not guarantee a diagnosis:

That would have saved me a lot of trouble if I'd believed myself. And it took ages, we went through about five or six neurologists /// Nothing. It was just dreadful because I kept thinking if I tell them what's wrong, what's happening and the symptoms, they're bound to know /// And nobody knew.

(participant with PCA)

A minority of participants (n=3) reported a timely and efficient diagnostic process, and this was mostly attributed to their own efforts towards information-seeking but for one gentleman was attributed to the 'pot-luck' of his GP's professional connections. The remaining dyads (n=17) described stress caused by having to persist in their search for a diagnosis for what they considered to be an unacceptably long period of time. This is consistent with existing literature which describes the benefits of and need to prioritise the early diagnosis of dementia for individuals, families and society[47-48], however this study highlights the particular barriers those with PCA face in receiving a timely diagnosis, owing to the rarity of the condition, associated lack of professional awareness and atypical symptom profile which lead them to exploring an eye-health route. Consistent with this idea

For peer review only - http://bmjopen.bmj.com/site/about/guidelines.xhtml

of the importance of having knowledge of the illness, many participants explicitly stated the relief they experienced when the diagnosis was provided (n=11).

Following diagnosis, there was a widely reported lack of accessible information. This has previously also been reported by those adjusting to a diagnosis of tAD[49-50] and young onset dementia specifically[51] and the current findings therefore add to knowledge about the varied types of dementia for which advice and information is needed. The rarity of PCA had the potential to be an ongoing source of stress over time in that those living with the diagnosis repeatedly found themselves better informed on the condition than health-care professionals they came into contact with, often having to re-explain the syndrome and their symptoms on multiple occasions.

Interacting with the physical environment

The nature of the symptoms (i.e. predominantly visual) meant effectively interacting with the physical environment was the predominant issue. This included interactions within and outside the home environment and with activities both functional and 'fun'.

Every participant described complications with completing self-care tasks, most prominently dressing and cooking. Difficulties with dressing included finding or selecting clothes or shoes, orienting them and using fastenings:

I do struggle a bit sometimes in working out which way round shirts go /// If it's all in a big heap, which it generally is, it's just a question of I will perhaps turn it round, sort of, two or three times before I work out where the collar is. (participant with PCA)

Dressing problems were exacerbated by distinguishing clean versus soiled clothes, seeing the closet and bedrooms being a shared space. Dressing assistance was frequently obtained from a family member or by decluttering, organising and simplifying the bedroom environment. In contrast to those with tAD, where problems with dressing may be attributable to problems with sequential task performance and attention[52], most of those with PCA articulately described clear visuo-percpetual and visuo-spatial processing problems underpinning their difficulties. Those with PCA were able to be similarly articulate about their choices and preferences around clothing and remained motivated to initiate dressing activities, which may be in contrast to those with tAD whose dressing may more commonly be disrupted by temporal disorientation and lack of motivation[53].

With cooking, typical problems were with locating ingredients/equipment in the kitchen, reading labels, following recipes, using appliances, or confidently and safely handling hot materials. For cooking or other household tasks, some individuals attempted to use visually salient strategies such

For peer review only - http://bmjopen.bmj.com/site/about/guidelines.xhtml

as labelling cupboards or putting a red dot on the start button of an appliance. The effectiveness of these strategies varied and more typically individuals retreated from these activities. As with dressing, when these difficulties are reported in the literature on tAD they are usually attributable to declining executive function skills[54].

Of particular importance to participants were difficulties they had in engaging with a wide range of hobbies and interests including reading, DIY, sports and arts activities. The impact of this seemed heightened by the typically young age of onset which saw all participants either approaching retirement age,having recently retired or reducing their working hours, and therefore allocating and looking forward to increasing time related to leisure activities:

I think we thought we'd be going out to theatre and travelling, and things more, whereas I'm planning in 2015 to make it the year I'm going to go to the matinees, try that. But, travelling has virtually stopped. We were going to take John's mother to the Christmas markets, thinking, well, with her help, I can probably get John on and off the train, but it... with her breaking her hip, that's another thing, another holiday went because of that, and so we thought, this is the time, this will be the time in our life that we need to travel, and this is the time in life, for one reason or another, we can't. /// Yes, I think we thought this would be the golden years. (family carer)

The stress at having to retreat from or renegotiate hobbies is consistent with a study which looked at aspects which are important for quality of life but challenged by dementia[55]. However the barriers here were largely due to the specific visuo-spatial and visuo-perceptual deficits, which is in contrast to a study by Giebel et al.[56] in which carers described those with tAD as having difficulties with the initiation rather than performance of such activities.

Beyond the home environment, all participants had difficulties navigating the external environment either on foot, by car or on public transport. Particular challenges were way- finding through crowds, reading signs or maps, general orientation, and using stairs/escalators:

I'm usually quite okay here but the minute I step outside the door it all goes mad. /// Life goes a bit strange, yes. /// even inside the village can be a bit strange but definitely when I go catching buses and dealing with... interacting with people, in general, no, it's not great, not great ./// I don't know what happens but it goes mad, yes. Not all the time and not every time but I'm much less comfortable and avoid, now, going unless I really need to go into town.

(participant with PCA)

Participants commonly stated they relied on routine responses to the environment and cues within it (e.g. using the same underground line or bus) or environmental cues (e.g. recognising a street by a

For peer review only - http://bmjopen.bmj.com/site/about/guidelines.xhtml

particular shop or church), but naturally the external environment is not a stable one. In the case of (e.g.) a disruption in public transportation or a lorry blocking the view of an environmental cue, problems arose such as the person with PCA getting lost or disoriented. It is important to note that for most this was not due to forgetfulness, distractibility or other executive function deficits as might be expected in cases of tAD[57-58], but because of problems accurately perceiving visual information about the environment which would help individuals to work out where they were in relation to their target destination.

The difficulties that participants described in interacting with their physical environments are compatible with the sorts of neuropsychological deficits documented in people with PCA in the literature. Deficits like visual crowding, simultanagnosia, spatial navigational problems and apraxia[4 9 14] corroborate with the issues people described with locating and manipulating objects, reading and dressing in this study.

As described above, the tendency towards simplification and familiarity meant withdrawing from certain activities. In addition though, almost all participants described ongoing uncertainty and unpredictability associated with the disease profile, commenting that the difficulties were not reliably ever-present (n=16). This uncertainty was once again exacerbated by a reported lack of disease-specific provision and guidance. A minority of participants opted for off-the-shelf adaptations for those with eye-health problems, for example a symbol cane (n=3), or had been in touch with the Royal National Institute for the Blind regarding visual aids (n=5) but most took a self-initiated, largely trial and error approach owing to the unusual, unpredictable and continually changing nature of the symptoms. This process was rife with uncertainty regarding if things would work, why they might not, and over time, how long they would continue to work:

Camilla can still read, so if it's just one word it's okay /// but we've done different colour coding [on shampoo/conditioner bottles] and this sort of stuff but then she forgets which colour's which. So it's not, you know, it seems to be simple…but then there'll be some other obstacle along the way.

(family carer)

This family carer provides an example of the complexity of progressive cognitive decline where strategies to compensate for the dominant visual symptoms may rely on other cognitive capacities (e.g. memory) which may also be affected to an extent. This was in contrast to those without memory impairment at the time of interview (n=9) who frequently relied on the familiarity of their environment to help them find their way or something they needed, often closing their eyes or feeling their way to minimise any confusing visual information.

For peer review only - http://bmjopen.bmj.com/site/about/guidelines.xhtml

Allen et al.[59] recently described a similar trial and error approach to environmental adaptations being employed by community-dwelling people with tAD and their carers, but these were more often triggered by – and designed to ameliorate – difficulties associated with dominant memory problems (e.g. using labels as reminders).

Acknowledging the temporal context, the participant above like many others referred to what his wife could 'still' do – appearing to demonstrate an anticipation of continuing decline over time. This is something commonly reported throughout the dementia literature in general[60-61]. In the case of PCA though, this was coupled with a relative paucity of accessible knowledge or professional guidance as to how the particular course of disease would progress.

Implications within the psychosocial environment

Overall, there were broader psychosocial ramifications arising from these day- to- day difficulties such as maintaining independence and contemplating an uncertain short and long term future.

The symptoms themselves and resulting difficulties in interactions within the physical environment naturally impacted individuals' ability to perform daily activities independently:

I think that is the worst thing I can do nothing for myself so all the time you've got to ask somebody

(participant with PCA)

Psychosocial strategies used to mediate the stress associated with performance of these daily activities were largely the provision of physical assistance and reallocation of responsibilities within the dyad or family. Over time this often resulted in increasing feelings of dependence:

One of my big problems is frustration that I can't do things I want to do. I don't need to do them but because I've always been able to do them it really irks me to have to phone my son up and say, come and put this together for me. (participant with PCA)

This example illustrates a common phenomenon reported in the interviews – the way this perceived dependency resulted in changing roles and responsibilities within families. The extent to which this caused secondary strain for individuals seemed to be mediated by contextual factors including the age of onset, personality factors and previous relationship quality. Interestingly, the one couple who were recently married (< 5 years) described their biggest challenges as being with organising the household responsibilities, though they were able to emotionally support each other well. This was perhaps owing to their less well-established household roles and responsibilities but their relatively recently established and continuing affection for each other.

For peer review only - http://bmjopen.bmj.com/site/about/guidelines.xhtml

With age of onset, individuals had often been at the peak of their careers and/or in a critical position within the family system (e.g. looking after both children and elderly parents). Such activities and the roles they represented were often defining in terms of individuals' senses of self and identity:

…he does all the ironing and everything now. And I say, no, that's my job. /// I'd always done it, you know. [He] was always working and I had the children and everything, and just did it. /// And I must admit, I sit here now and I think, I can't do anything. (participant with PCA)

The threat that dementia and the associated decline in functioning can pose for a person's sense of identity and independence is well documented throughout the existing literature, even when functional capacity is disrupted by memory problems[61-62] rather than predominant cortical visual deficits as seen here.

Family carers (n=13) also described concerns they had about their competencies in taking on the new role of caregiver and reported the strain of uncertainties in knowing how and when to provide help. Many noted trying to strike a balance between getting things done, preventing their family member's distress or frustration, and encouraging or facilitating their ongoing independence.

Any stress and strain for participants who had a diagnosis of PCA regarding the impact on identity, role and independence were arguably emphasised by their relatively intact long-term memory functions and abilities and inclination to reflect on and compare their previous experiences with their current situations. One way this caused stress was that the person with PCA could reflect on themselves and their declining abilities and in doing so most (n=15) were concerned that they were becoming a burden to their partner or wider family. However, on the other hand, these relatively intact capacities were also able to contribute to relieving stress for the majority of couples who took an interdependent or 'teamwork' (n=16) approach to managing the difficulties, via continued collaboration and joint problem solving. Those dyads who didn't adopt such a team-based approach (n=4) appeared not to because of either factors relating to: their previous relationship quality (e.g. living fairly independent lives); personality (e.g. pride, stubbornness); or the cognitive decline of the person with PCA (e.g. poor memory for shared events). The dominant 'teamwork' approach or sense of 'being on the same page' and navigating the illness experience together was made evident in shared story-telling during the joint interviews and in the corresponding and complimentary accounts given in the individual interviews However there were also some instances of discrepancies in the accounts of the person with PCA and their family carer. Sometimes differing recollections of events (e.g. a family occasion, first symptom onset) would seem to be easily explained by the person with PCA's concurrent memory impairment, but in other cases the discrepancies were harder to

For peer review only - http://bmjopen.bmj.com/site/about/guidelines.xhtml

unpick. Several dyads offered differing accounts of the person with PCA's functional ability. In one case, a daughter attested that her mother was trying to not let us, the researchers, know how impaired she was by insisting she was still doing household chores, and that the discrepancy could be explained as an exercise in self-presentation. In another case, a man with PCA and his wife disagreed over whether he could safely go for a walk unattended – she considered his wayfinding abilities to be too compromised and he considered her to be too closely monitoring him and disproportionately concerned. Day-to-day difficulties were also attributed to different underlying symptoms, for example one man with PCA put his problem with navigating the stairwell at a relative's house down to the area being 'dim' and 'dark', whereas his wife put this down to him not remembering where he was correctly:

… you still get lost on that landing. /// So it's a square area with four closed doors where does he go? And he never knows…like where is it, where on earth is it? So he can't retain the information…this is like every single time…he is walking down those steps for the first time. (family carer)

These discrepancies – though not the common pattern in the current study – clearly highlight the importance of acknowledging potential differences in perspectives and the challenges these could pose in the day-to-day understanding of and responses to the symptom profile and its impact.

Despite these occasional discrepancies, many individuals with PCA (n=11) expressed extensive gratitude or feeling 'lucky' for their spouse/family carer while also normalising any symptom-specific dependency as one of many ways in which they and their family member worked together to manage life's challenges:

If you get married, sickness and in health, you have to keep to these things… I'm old-fashioned enough to think... you know, if it were me, he would look after me, I have no doubt. /// So, you know, what I think, and this is what I say to him, we're married, we're two parts of a whole, so in many ways it affects me, because then when you are supposed to be as one, as a whole…then you have to look after the other half of you, and, you know, by keeping one half healthy, helps the other half.

(family carer)

There was a suggestion that a relatively preserved insight and ability to plan in people with PCA promoted a continuation of closeness and collaboration between dyads, potentially contrasting with previous studies involving participants with young onset- but memory-led, dementias. Baikie[63] reported a loss of joint decision making in marital relationships while separateness made up part of an overarching theme in a study by O'Shaughnessy et al.[64] about the impact of dementia on the marital relationship. Similarly, Wright[65] described how a lack of awareness contributed to

For peer review only - http://bmjopen.bmj.com/site/about/guidelines.xhtml

discrepancies in the accounts of people with dementia and their spouses regarding their experiences of tension within the relationship and also an overall reduction in shared meanings made about the illness experience. However, there are also reports of couples living with memory-led dementias taking a continued team-work approach[49] and sustained reciprocity in consideration of the others' needs[66], both of which serve as a useful reminder of our need to interpret with caution. An example of this within our own study was one of the male participants who demonstrated concurrent memory problems along with his dominant visual processing deficits, whose wife commented:

Everything's more flat, yes. And he's got no sense of time, so whereas before… he would go…oh yes…this is the year that she said was our silver wedding anniversary ///now he's got to rely on the children to say…we'd better do something about it. /// I found it painful. (family carer)

Two points seem salient here. First, the dementias progress in different ways for different people. And secondly, given the variation, states of separateness or connectedness, or team-work versus independence, do not apply discretely and exclusively to a group of people with one diagnosis and not to another. The participants with PCA interviewed here will likely progress to have more memory challenges over time, potentially posing additional challenges to shared meaning-making, and those in the early stages of more typical dementias are increasingly shown to be able to reflect reliably and accurately on their own experiences and abilities[66-67], something essential for joint problem solving and shared decision-making.

Another factor to consider is the sociocultural context within which research questions are framed and studies carried out. As the value put on quality of life surpasses that of quantity and as cures for dementias remain elusive, attention has shifted to ideas of preserving personhood of those with dementia and more recently still, the couple-hood of dyads living with a diagnosis. Acknowledging the person and not just their disease has also meant a shift in focus from seeking to document deficits, loss and failings and attempts to capture the whole breadth of experience as attempted here – the positives, strengths, closeness and resilience[68-69].

One day at a time

Beyond diagnosis and in terms of longer term ongoing coping with the diagnosis, uncertainty and a lack of knowledge persisted, this time with regards to what to expect and how the disease would progress:

For peer review only - http://bmjopen.bmj.com/site/about/guidelines.xhtml

There's little point in thinking about the future because, in that sense, one would have to have a model of what the future may hold and therein lies part of the difficulty, that I can't map that future and make any choices. (participant with PCA)

This impacted how able many dyads felt to effectively plan for the future and many described taking a conscious decision to not think about it, given that there seemed to be little purpose or pleasure in doing so because of the incurable and progressive nature of the diagnosis:

Every now and again I get down, mainly because if I think too long about what the future holds then … it's counter-productive /// It's going to happen. There's nothing you can do about it. You know, it's like one of those things. It's nothing... it's all... you can give a problem a lot of thought if there's an answer; right, do we do this or do we do that? Right, think about it a long time, perhaps worry about it for a couple of days. Right, let's do that. With this, there isn't... What's Plan B? You haven't got a Plan B. And that's this situation. There isn't a Plan B. (participant with PCA)

In shifting the focus away from an uncertain future, many described their approach as being centred around ideas of 'keeping going' and 'getting on with things', with almost all dyads describing efforts to maintain normality as far as possible (n=17):

When she [my wife] got her head around what she had, she said, there's nothing I can do about it, we've just got to get on with it. And we just carried on as normal. /// When it crops up, I deal with it, but 99% of the time, we just carry on as normal. //// I mean, obviously you gradually get worse and worse, but, you know. (family carer)

This family carer's comment clearly demonstrates the complexity and significance of temporality in describing the simultaneous day-by-day approach that can exist in combination with broader acknowledgement and anticipation of ongoing decline over the long term.

There were several reports of professionals endorsing or echoing this approach of living in the moment:

She [doctor] just looked at him and... put her hands on his legs and said, just live your life /// just go on and live your life, that's all you can do. (family carer)

This comment also seemed to address the inevitability of the progression of the disease in describing taking this approach as the only thing participants could do in the face of the diagnosis (i.e. in the

For peer review only - http://bmjopen.bmj.com/site/about/guidelines.xhtml

absence of a cure). Perhaps it also hints at the lack of published guidance and knowledge about progression of the disease and what to expect which may have enabled or assisted longer term care planning and management. This is corroborated by a recent paper which identified the challenges families and practitioners face in finding tailored, disease-specific information and practical advice about PCA which is evidence-based[70].

Taking a day-by-day approach was not only preferred but required due to the ever-changing nature of the symptom profile as the disease progressed. Individuals and families were continually responsive and the majority described being attuned to the necessary ongoing adjustments and adaptations required by the continual change that is characteristic of the disease profile (n=16):

Yes, as I say, if I let my mind go there [the future], I will probably collapse in a heap, so I find it's best just to deal with things as they present, and just try and think one step ahead, and not too far, because, as I keep being told, every individual with the disease is different, and they can make no...they've got no crystal balls to see into the future, about exactly how it's going to pan for any...one person (family carer)

The sense of needing to balance the maintenance of normality in the face of diagnosis-related changes which require ongoing adjustment is also commonly reported throughout the qualitative literature on dementia[49 64 67 69]. A decision to focus on the 'here and now' is also widely reported, owing to the uncertainty or discomfort associated with thoughts about the future, in line with what was reported here[49 64]. These similarities with existing literature perhaps highlight the progressive nature which is common to all variations of AD and dementias more broadly.

5. Discussion

This study has illustrated some of the diagnosis-specific stressors associated with PCA and the various ways individuals and families attempt to mediate these. The characteristic visually-dominated symptom profile led to primary difficulties in interacting with the physical environment. These were situated within a complex psychosocial environment involving a range of roles and responsibilities requiring reallocation and various individuals' longstanding preferences regarding independence versus asking for, accepting and providing help. The rarity of the condition meant a lack of knowledge and accessible information about the symptoms, disease course and provision of support for those living with PCA and healthcare professionals all of which contributed to stress. The

For peer review only - http://bmjopen.bmj.com/site/about/guidelines.xhtml

temporal context was of particular significance in shaping the stress process in terms of the time of life (e.g. employment/retirement status; position in the family); previous levels of performance and engagement in activities/interests; consideration of and concern about the future; and the time-limited efficacy of support strategies.

This study offers an original contribution in looking at the day-to-day impacts of progressive visual impairment related to dementia rather than the more typically dominant memory loss[71]. Also, the inductive, qualitative methodology used here offers unique insights to complement the existing PCA literature which is largely laboratory-based and concentrated on specifying the cognitive profile and underlying pathology of the disease. This study has gleaned insights firmly grounded in participants' day-to-day experiences, within their home environments and recounted in their own words. This has allowed the full range of experience to be reported and documented. For example, families were able to share the problematic nature of symptoms and ongoing decline, but also their resilience in their collaborative and creative approaches to developing coping strategies and in continually adapting to the diagnosis-related changes.

Although this is the first qualitative study of PCA the findings here bear relevance to existing literature in several ways. The stress caused by the uncertainty and atypical nature of the diagnosis and the impact of lacking disease-specific support and guidance echoes that which other authors have described in relation to other rare diseases[72]. The findings here may also bear relevance for those living with or seeking diagnoses of other rarer types of dementias, into which research is rightfully ever-increasing (e.g. behavioural variant frontotemporal dementia and the primary progressive aphasias[73]).

Overall, we need to remain critical of the questions we ask and sensitive to the individual differences in disease profile across and within diagnostic groups – especially in terms of what this means in terms of psychosocial impact for the individual and family. Perhaps the most significant contribution of this paper is in outlining the different mechanisms (e.g. visual versus memory problems) which can underpin difficulties with daily activities – even if the psychosocial ramifications of such difficulties are similar or overlapping. Understanding the ways in which the experiences of those with different diagnoses and at different stages of their disease overlap and diverge will be essential if we are to build a knowledge base in which all the complex stories of living with the dementias are told.

A strength of the study is that those with a diagnosis of PCA and a family member were interviewed together and separately, ensuring that multiple perspectives were represented in the data set. In

For peer review only - http://bmjopen.bmj.com/site/about/guidelines.xhtml

this case, and in contrast to some existing literature on spousal couples living with tAD[63-65] the relatively well preserved insight of those with PCA resulted in largely congruent accounts of both parties in terms of levels of abilities and shared understandings of the illness experience. However, the discrepancies discussed above – and the varying plausible reasons for them – highlight the importance of interviewing in such a way that rich data from a range of perspectives can be gleaned and also the sensitivities and ethics around the assigning of credit to and/or interpretation of such data. That interviews were conducted in the home environment enriched the data in permitting researchers an in-depth understanding of the everyday physical environment in which difficulties emerged and were responded to and often acted as a useful prompt for participants when discussing challenges and strategies. Working within a process model encouraged consideration of related underlying mechanisms, resulting stressors and responsive coping strategies. The study also makes a broader contribution in highlighting a potential limitation of the Stress Process Model in not taking account of the physical environment as a potential source or mediator of stress, despite suggestions that this may play a particularly significant role for people with PCA[74] and dementia in general[75-77]. A possible limitation of the study is that the home-based nature of the interviews may have deterred dyads who were not managing well from taking part, and as such the findings may not capture the full range of coping responses to the stress process. The interviews took place at one time point, and the emergent importance of the temporal context may make this another limitation of the study.

In light of these findings, implications for clinicians centre around the need for increased knowledge and provision of information – particularly in a diagnostic context – which is particular to the challenges associated with dementia-related visual impairment and sensitive to the psychosocial ramifications of these difficulties. In addition, dominant difficulties interacting with the physical world may make those with PCA particularly suitable for psychosocial interventions targeted at the marital or family unit as a whole, owing to the relative cognitive strengths of those with PCA (e.g. insight, memory and language). Unanswered questions remain about how PCA progresses beyond the moderate stages and also how visual problems may affect people with more typical, memory-led forms of dementia, perhaps at a later stage when they are less easily communicated. Equally, future focussed work which factors in the impacts of secondary impairments such as the concurrent memory and language impairments seen in subgroups of our sample here would be helpful to further unpick and illustrate the complexity of the PCA syndrome and the varying impacts it can have. Future research that looks at this both over time and taking account of the multiple perspectives inherent in any dementia journey would constitute valuable and original contributions to knowledge.

For peer review only - http://bmjopen.bmj.com/site/about/guidelines.xhtml

6. Conclusion

This study provides new insights into the stress process for individuals and families living with PCA, from the search for a diagnosis through to the daily challenges of living with dementia-related visual impairment. Increased availability and accessibility of information about PCA, its early symptoms and progression for both healthcare professionals and affected families would be beneficial in aiding timely diagnosis and minimising ongoing stress and uncertainty. Key considerations in the design of supportive interventions for those with PCA would be timeliness and sensitivity to the complexities of the surrounding psychosocial environment within which they must be adopted and adapted to over time.

7. Acknowledgements

We would like to thank all those who participated in this study. This work was supported by Economic and Social Research Council (ESRC)-National Institute for Health Research (NIHR) (UK) grant ES/L001810/1. Grant title: Seeing what they see: compensating for cortical visual dysfunction in Alzheimer's Disease. SC is also supported by the NIHR Queen Square Dementia Biomedical Research Unit and an Alzheimer's Research UK Senior Research Fellowship. RW is supported on a Brunel University London doctoral scholarship.

8. Conflict of Interest

None.

9. Data Statement

Data will be made available in accordance with funder guidelines after the completion of the project (March 2018).

10. Author Statement

EH contributions to study protocol development; data collection, analysis and interpretation; manuscript preparation (drafting and incorporation of comments/amendments).

MPS contributions to study protocol development; data collection, analysis and interpretation; provided comments on draft manuscripts and approved final manuscript.

RW contributions to study protocol development; data collection; provided comments on draft manuscript.

For peer review only - http://bmjopen.bmj.com/site/about/guidelines.xhtml

KY contributions to study protocol development; recruitment; data collection, analysis and interpretation; provided comments on draft manuscript.

AM contributions to study protocol development; data collection, analysis and interpretation.

MG contributions to study protocol development; provided comments on draft manuscript.

KG contributions to study protocol development; comments on draft manuscript.

SC contributions to study protocol development; provided comments on draft manuscripts and approved final manuscript.

11. References

References

1. Alzheimer's Society. *Dementia UK: Update. Second edition.* Alzheimer's Society, London. 2014.
2. Alzheimer's Society. *What is young-onset dementia?* [Fact sheet]. Available from https://www.alzheimers.org.uk/site/scripts/download_info.php?fileID=17662015. 2015.
3. Crutch SJ, Schott JM, Rabinovici GD, Murray M, Snowden JS, van der Flier WM, Dickerson BC, Vandenberghe R, Ahmed S, Bak TH, Boeve BF. Consensus classification of posterior cortical atrophy. *Alzheimer's & Dementia.* 2017 Mar 2.
4. Tang-Wai DF, Graff-Radford NR, Boeve BF, Dickson DW, Parisi JE, Crook R, Caselli RJ, Knopman DS, Petersen RC. Clinical, genetic, and neuropathologic characteristics of posterior cortical atrophy. *Neurology*. 2004 Oct 12;63(7):1168-74. Available from: http://dx.doi.org/10.1212/01.wnl.0000140289.18472.15
5. Renner JA, Burns JM, Hou CE, McKeel DW, Storandt M, Morris JC. Progressive posterior cortical dysfunction A clinicopathologic series. *Neurology*. 2004 Oct 12;63(7):1175-80. Available from: http://dx.doi.org/10.1212/01.wnl.0000140290.80962.bf
6. Crutch SJ, Lehmann M, Schott JM, Rabinovici GD, Rossor MN, Fox NC. Posterior cortical atrophy. *The Lancet Neurology*. 2012 Feb 29;11(2):170-8. Available from: http://dx.doi.org/10.1016/s1474-4422(11)70289-7
7. Crutch SJ, Lehmann M, Warren JD, Rohrer JD. The language profile of posterior cortical atrophy. *Journal of Neurology Neurosurgery & Psychiatry*. 2012 Nov 1:jnnp-2012. Available from: http://dx.doi.org/10.1136/jnnp-2012-303309
8. Ahmed S, Baker I, Husain M, Thompson S, Kipps C, Hornberger M, Hodges JR, Butler CR. Memory impairment at initial clinical presentation in posterior cortical atrophy. *Journal of*

For peer review only - http://bmjopen.bmj.com/site/about/guidelines.xhtml

Alzheimer's Disease. 2016 Jan 1;52(4):1245-50. Available from: http://dx.doi.org/10.3233/jad-160018

9. Mendez MF, Ghajarania M, Perryman KM. Posterior cortical atrophy: clinical characteristics and differences compared to Alzheimer's disease. *Dementia and Geriatric Cognitive Disorders.* 2002;14(1):33-40. Available from: http://dx.doi.org/10.1159/000058331
10. Victoroff J, Ross GW, Benson DF, Verity MA, Vinters HV. Posterior cortical atrophy: neuropathologic correlations. *Archives of Neurology*. 1994 Mar 1;51(3):269-74.
11. Lehmann M, Crutch SJ, Ridgway GR, Ridha BH, Barnes J, Warrington EK, Rossor MN, Fox NC. Cortical thickness and voxel-based morphometry in posterior cortical atrophy and typical Alzheimer's disease. *Neurobiology of Aging*. 2011 Aug 31;32(8):1466-76.
12. Charles RF, Hillis AE. Posterior cortical atrophy: clinical presentation and cognitive deficits compared to Alzheimer's disease. *Behavioural Neurology*. 2005 Jan 1;16(1):15-23.
13. Yong KX, Shakespeare TJ, Cash D, Henley SM, Warren JD, Crutch SJ. (Con) text-specific effects of visual dysfunction on reading in posterior cortical atrophy. *Cortex*. 2014 Aug 31;57:92-106. Available from: http://dx.doi.org/10.1016/j.cortex.2014.03.010
14. Yong KX, Shakespeare TJ, Cash D, Henley SM, Nicholas JM, Ridgway GR, Golden HL, Warrington EK, Carton AM, Kaski D, Schott JM. Prominent effects and neural correlates of visual crowding in a neurodegenerative disease population. *Brain*. 2014 Oct 28;137(12):3284-99. Available from: http://dx.doi.org/10.1093/brain/awu293
15. Shakespeare TJ, Yong KX, Foxe D, Hodges J, Crutch SJ. Pronounced impairment of everyday skills and self-care in posterior cortical atrophy. *Journal of Alzheimer's Disease*. 2015 Jan 1;43(2):381-4. Available from: http://dx.doi.org/10.1016/j.jalz.2014.05.427
16. Suárez-González A, Crutch SJ, Franco-Macías E, Gil-Néciga E. Neuropsychiatric symptoms in Posterior cortical atrophy and Alzheimer disease. *Journal of Geriatric Psychiatry and Neurology*. 2016 Mar;29(2):65-71. Available from: http://dx.doi.org/10.1177/0891988715606229
17. Kasl-Godley J, Gatz M. Psychosocial interventions for individuals with dementia: an integration of theory, therapy, and a clinical understanding of dementia. *Clinical Psychology Review*. 2000 Aug 31;20(6):755-82. Available from: http://dx.doi.org/10.1016/s0272-7358(99)00062-8
18. Cahill S, Woods B, Droes RM, Husband H, Burnham M, Urbas S, Charlesworth G, Franco M, Cantegreil-Kallen I, Clare L, Vernooij-Dassen M. *Early psychosocial interventions in dementia: evidence-based practice*. London: Jessica Kingsley Publishers; 2008 Nov 15.

For peer review only

19. Gilhooly KJ, Gilhooly ML, Sullivan MP, McIntyre A, Wilson L, Harding E, Woodbridge R, Crutch S. A meta-review of stress, coping and interventions in dementia and dementia caregiving. *BMC Geriatrics*. 2016 May 18;16(1):106. Available from: http://dx.doi.org/10.1186/s12877-016-0280-8
20. Bakker C, de Vugt ME, Vernooij-Dassen M, van Vliet D, Verhey FR, Koopmans RT. Needs in early onset dementia: a qualitative case from the NeedYD study. *American Journal of Alzheimer's Disease & Other Dementias*. 2010 Dec;25(8):634-40. Available from: http://dx.doi.org/10.1177/1533317510385811
21. Johannessen A, Möller A. Experiences of persons with early-onset dementia in everyday life: a qualitative study. *Dementia*. 2013 Jul;12(4):410-24. Available from: http://dx.doi.org/10.1177/1471301211430647
22. Griffin J, Oyebode JR, Allen J. Living with a diagnosis of behavioural-variant frontotemporal dementia: The person's experience. *Dementia*. 2016 Nov;15(6):1622-42. Available from: http://dx.doi.org/10.1177/1471301214568164
23. Paxton JL, Peavy GM, Jenkins C, Rice VA, Heindel WC, Salmon DP. Deterioration of visual-perceptual organization ability in Alzheimer's disease. *Cortex*. 2007 Dec 31;43(7):967-75.
24. Allen AP, Curran EA, Duggan Á, Cryan JF, Chorcoráin AN, Dinan TG, Molloy DW, Kearney PM, Clarke G. A systematic review of the psychobiological burden of informal caregiving for patients with dementia: Focus on cognitive and biological markers of chronic stress. *Neuroscience & Biobehavioral Reviews*. 2017 Feb 1;73:123-64. Available from: http://dx.doi.org/10.1016/j.neubiorev.2016.12.006
25. Haley WE, Levine EG, Brown SL, Bartolucci AA. Stress, appraisal, coping, and social support as predictors of adaptational outcome among dementia caregivers. *Psychology and Aging*. 1987 Dec;2(4):323-30. Available from: http://dx.doi.org/10.1037/0882-7974.2.4.323
26. Kramer BJ, Vitaliano PP. Coping: A review of the theoretical frameworks and the measures used among caregivers of individuals with dementia. *Journal of Gerontological Social Work*. 1995 Apr 7;23(1-2):151-74.
27. Norman I, Redfern S, Briggs K, Askham J. Perceptions and management of change by people with dementia and their carers living at home. *Dementia*. 2004 Feb;3(1):19-44. Available from: http://dx.doi.org/10.1177/1471301204032614
28. Borden W. Stress, coping, and adaptation in spouses of older adults with chronic dementia. In *Social Work Research and Abstracts 1991 Mar 1* (Vol. 27, No. 1, pp. 14-21). Oxford University Press. Available from: http://dx.doi.org/10.1093/swra/27.1.14

For peer review only

For peer review only - http://bmjopen.bmj.com/site/about/guidelines.xhtml

29. Kim Y, Schulz R. Family caregivers' strains: comparative analysis of cancer caregiving with dementia, diabetes, and frail elderly caregiving. *Journal of Aging and Health*. 2008 Aug;20(5):483-503.

30. Kneebone II, Martin PR. Coping and caregivers of people with dementia. *British Journal of Health Psychology*. 2003 Feb 1;8(1):1-7. Available from: http://dx.doi.org/10.1348/135910703762879174

31. Li R, Cooper C, Bradley J, Shulman A, Livingston G. Coping strategies and psychological morbidity in family carers of people with dementia: a systematic review and meta-analysis. *Journal of Affective Disorders*. 2012 Jun 30;139(1):1-1. Available from: http://dx.doi.org/10.1016/j.jad.2011.05.055

32. Pearlin LI, Mullan JT, Semple SJ, Skaff MM. Caregiving and the stress process: An overview of concepts and their measures. *The Gerontologist*. 1990 Oct 1;30(5):583-94. Available from: http://dx.doi.org/10.1093/geront/30.5.583

33. Judge KS, Menne HL, Whitlatch CJ. Stress process model for individuals with dementia. *The Gerontologist.* 2009 Dec 18;50(3):294-302. Available from: http://dx.doi.org/10.1093/geront/gnp162

34. Rubin, HJ, Rubin, IS. *Qualitative interviewing: The art of hearing data*. London: Sage; 2011.

35. Taylor B, de Vocht H. Interviewing separately or as couples? Considerations of authenticity of method. *Qualitative Health Research*. 2011 Nov;21(11):1576-87. Available from: http://dx.doi.org/10.1177/1049732311415288

36. Morgan DL, Ataie J, Carder P, Hoffman K. Introducing dyadic interviews as a method for collecting qualitative data. *Qualitative Health Research*. 2013 Sep;23(9):1276-84. Available from: http://dx.doi.org/10.1177/1049732313501889

37. Emmel, N., & Clark, A. The methods used in connected lives: Investigating networks, neighbourhoods and communities. NCRM Working Paper. *National Centre for Research Methods.* 2009.

38. Braun V, Clarke V. Using thematic analysis in psychology. *Qualitative Research in Psychology*. 2006 Jan 1;3(2):77-101. Available from: http://dx.doi.org/10.1191/1478088706qp063oa

39. Morse JM, Barrett M, Mayan M, Olson K, Spiers J. Verification strategies for establishing reliability and validity in qualitative research. *International Journal of Qualitative Methods*. 2002 Jun;1(2):13-22. Available from: http://dx.doi.org/10.1177/160940690200100202

40. Sandelowski M. Rigor or rigor mortis: the problem of rigor in qualitative research revisited. *Advances in Nursing Science*. 1993 Dec 1;16(2):1-8. Available from: http://dx.doi.org/10.1097/00012272-199312000-00002

For peer review only

For peer review only - http://bmjopen.bmj.com/site/about/guidelines.xhtml

41. Harvey L. Beyond member-checking: A dialogic approach to the research interview. *International Journal of Research & Method in Education*. 2015 Jan 2;38(1):23-38. Available from: http://dx.doi.org/10.1080/1743727x.2014.914487
42. Tong A, Sainsbury P, Craig J. Consolidated criteria for reporting qualitative research (COREQ): a 32-item checklist for interviews and focus groups. *International Journal for Quality in Health Care*. 2007 Dec 1;19(6):349-57. Available from: http://dx.doi.org/10.1093/intqhc/mzm042
43. Warrington, EK. *The Camden memory tests: Manual* (Vol. 1). Hove: Psychology Press; 1996.
44. K. Warrington Pat McKenna Lisa Orpwood E. Single word comprehension: a concrete and abstract word synonym test. *Neuropsychological Rehabilitation*. 1998 Apr 1;8(2):143-54. Available from: http://dx.doi.org/10.1080/713755564
45. Warrington, EK, James, M. *The visual object and space perception battery*. Bury St Edmunds: Thames Valley Test Company; 1991.
46. Barnes J, Dickerson BC, Frost C, Jiskoot LC, Wolk D, van der Flier WM. Alzheimer's disease first symptoms are age dependent: evidence from the NACC dataset. *Alzheimer's & Dementia*. 2015 Nov 30;11(11):1349-57.
47. Leifer BP. Early diagnosis of Alzheimer's disease: clinical and economic benefits. *Journal of the American Geriatrics Society*. 2003 May 1;51(5s2).
48. Robinson SM, Canavan M, O'Keeffe ST. Preferences of older people for early diagnosis and disclosure of Alzheimer's disease (AD) before and after considering potential risks and benefits. *Archives of Gerontology and Geriatrics*. 2014 Dec 31;59(3):607-12.
49. Bunn F, Goodman C, Sworn K, Rait G, Brayne C, Robinson L, McNeilly E, Iliffe S. Psychosocial factors that shape patient and carer experiences of dementia diagnosis and treatment: a systematic review of qualitative studies. *PLoS Medicine*. 2012 Oct 30;9(10):e1001331. Available from: http://dx.doi.org/10.1371/journal.pmed.1001331
50. Clemerson G, Walsh S, Isaac C. Towards living well with young onset dementia: An exploration of coping from the perspective of those diagnosed. *Dementia*. 2014 Jul;13(4):451-66. Available from: http://dx.doi.org/10.1177/1471301212474149
51. Wawrziczny E, Pasquier F, Ducharme F, Kergoat MJ, Antoine P. From 'needing to know'to 'needing not to know more': an interpretative phenomenological analysis of couples' experiences with early-onset Alzheimer's disease. *Scandinavian Journal of Caring Sciences*. 2016 Dec 1;30(4):695-703.

For peer review only - http://bmjopen.bmj.com/site/about/guidelines.xhtml

52. Namazi KH, Johnson BD. Dressing independently: A closet modification model for Alzheimer's disease patients. *American Journal of Alzheimer's Care and Related Disorders & Research*. 1992 Jan;7(1):22-8.
53. Teri L, Borson S, Kiyak HA, Yamagishi M. Behavioral disturbance, cognitive dysfunction, and functional skill: Prevalence and relationship in Alzheimer's disease. *Journal of the American Geriatrics Society*. 1989 Feb.
54. Perry RJ, Hodges JR. Attention and executive deficits in Alzheimer's disease: A critical review. *Brain*. 1999 Mar 1;122(3):383-404.
55. Thorgrimsen L, Selwood A, Spector A, Royan L, de Madariaga Lopez M, Woods RT, Orrell M. Whose quality of life is it anyway?: The validity and reliability of the Quality of Life-Alzheimer's Disease (QoL-AD) scale. *Alzheimer Disease & Associated Disorders*. 2003 Oct 1;17(4):201-8. Available from: http://dx.doi.org/10.1097/00002093-200310000-00002
56. Giebel CM, Challis DJ, Montaldi D. A revised interview for deterioration in daily living activities in dementia reveals the relationship between social activities and well-being. *Dementia*. 2016 Sep;15(5):1068-81. Available from: http://dx.doi.org/10.1177/1471301214553614
57. Chiu YC, Algase D, Whall A, Liang J, Liu HC, Lin KN, Wang PN. Getting lost: Directed attention and executive functions in early Alzheimer's disease patients. *Dementia and Geriatric Cognitive Disorders.* 2004;17(3):174-80. Available from: http://dx.doi.org/10.1159/000076353
58. Pai MC, Jacobs WJ. Topographical disorientation in community-residing patients with Alzheimer's disease. *International Journal of Geriatric Psychiatry*. 2004 Mar 1;19(3):250-5.
59. Allen F, Cain R, Meyer C. How people with dementia and their carers adapt their homes. A qualitative study. *Dementia*. 2017 Jan 1:1471301217712294. Available from: http://dx.doi.org/10.1177/1471301217712294
60. Roach P, Keady J, Bee P, Hope K. Subjective experiences of younger people with dementia and their families: implications for UK research, policy and practice. *Reviews in Clinical Gerontology*. 2008 May;18(2):165-74. Available from: http://dx.doi.org/10.1017/s0959259809002779
61. Harris PB, Keady J. Selfhood in younger onset dementia: transitions and testimonies. *Aging and Mental Health*. 2009 May 1;13(3):437-44. Available from: http://dx.doi.org/10.1080/13607860802534609

For peer review only - http://bmjopen.bmj.com/site/about/guidelines.xhtml

62. Clare L, Rowlands J, Bruce E, Surr C, Downs M. 'I don't do like I used to do': A grounded theory approach to conceptualising awareness in people with moderate to severe dementia living in long-term care. *Social Science & Medicine*. 2008 Jun 30;66(11):2366-77.
63. Baikie E. The impact of dementia on marital relationships. *Sexual and Relationship Therapy*. 2002 Aug 1;17(3):289-99. Available from: http://dx.doi.org/10.1080/14681990220149095
64. O'shaughnessy M, Lee K, Lintern T. Changes in the couple relationship in dementia care: Spouse carers' experiences. *Dementia*. 2010 May;9(2):237-58.
65. Wright LK. The impact of Alzheimer's disease on the marital relationship. *The Gerontologist*. 1991 Apr 1;31(2):224-37.
66. Merrick K, Camic PM, O'Shaughnessy M. Couples constructing their experiences of dementia: A relational perspective. *Dementia*. 2016 Jan;15(1):34-50. Available from: http://dx.doi.org/10.1177/1471301213513029
67. Menne HL, Kinney JM, Morhardt DJ. 'Trying to Continue to Do as Much as They Can Do' Theoretical insights regarding continuity and meaning making in the face of dementia. *Dementia*. 2002 Nov;1(3):367-82. Available from: http://dx.doi.org/10.1177/147130120200100308
68. Hellström I, Nolan M, Lundh U. Sustaining couplehood' Spouses' strategies for living positively with dementia. *Dementia*. 2007 Aug;6(3):383-409.
69. Molyneaux VJ, Butchard S, Simpson J, Murray C. The co-construction of couplehood in dementia. *Dementia*. 2012 Jul;11(4):483-502. Available from: http://dx.doi.org/10.1177/1471301211421070
70. Wilson, L., Gilhooly, M. L. M., Sullivan, M. P., Gilhooly, K. J., McIntyre, A., Woodbridge, R., Harding, E., Walton, J., & Crutch, S. Posterior cortical atrophy: resources and information. *Journal of Dementia Care.* 2016 Jan 1; 24(1): 32-34.
71. Steeman E, Casterlé D, Dierckx B, Godderis J, Grypdonck M. Living with early-stage dementia: A review of qualitative studies. *Journal of Advanced Nursing*. 2006 Jun 1;54(6):722-38. Available from: http://dx.doi.org/10.1111/j.1365-2648.2006.03874.x
72. Wallenius E, Möller K, Berglund B. Everyday impact of having a rare diagnosis. A questionnaire study. *Nordic Journal of Nursing Research*. 2009 Sep;29(3):13-7.
73. Ritchie K, Lovestone S. The dementias. *The Lancet*. 2002 Nov 30;360(9347):1759-66. Available from: http://dx.doi.org/10.1016/s0140-6736(02)11667-9
74. Tang-Wai DF, Lake A, Graff-Radford N. Posterior cortical atrophy. *Dementia: Comprehensive Principles and Practices*. 2014 Aug 1. Available from: http://dx.doi.org/10.1093/med/9780199928453.003.0010

For peer review only

75. O'Malley L, Croucher K. Housing and dementia care–a scoping review of the literature. *Health & Social Care in the Community*. 2005 Nov 1;13(6):570-7. Available from: http://dx.doi.org/10.1111/j.1365-2524.2005.00588.x

76. Fleming R, Purandare N. Long-term care for people with dementia: environmental design guidelines. *International Psychogeriatrics*. 2010 Nov;22(7):1084-96. Available from: http://dx.doi.org/10.1017/s1041610210000438

77. Woodbridge R, Sullivan MP, Harding E, Crutch S, Gilhooly KJ, Gilhooly ML, McIntyre A, Wilson L. Use of the physical environment to support everyday activities for people with dementia: A systematic review. *Dementia*. 2016 Aug 4: Available from: http://dx.doi.org/10.1177/1471301216648670

For peer review only

For peer review only - http://bmjopen.bmj.com/site/about/guidelines.xhtml

COREQ (COnsolidated criteria for REporting Qualitative research) Checklist

A checklist of items that should be included in reports of qualitative research. You must report the page number in your manuscript where you consider each of the items listed in this checklist. If you have not included this information, either revise your manuscript accordingly before submitting or note N/A.

Topic	Item No.	Guide Questions/Description	Reported on Page No.
**Domain 1: Research team and reflexivity**			
Personal characteristics			
Interviewer/facilitator	1	Which author/s conducted the interview or focus group?	6
Credentials	2	What were the researcher's credentials? E.g. PhD, MD	1
Occupation	3	What was their occupation at the time of the study?	-
Gender	4	Was the researcher male or female?	1
Experience and training	5	What experience or training did the researcher have?	1
Relationship with participants			
Relationship established	6	Was a relationship established prior to study commencement?	5
Participant knowledge of the interviewer	7	What did the participants know about the researcher? e.g. personal goals, reasons for doing the research	5-6
Interviewer characteristics	8	What characteristics were reported about the inter viewer/facilitator? e.g. Bias, assumptions, reasons and interests in the research topic	-
**Domain 2: Study design**			
Theoretical framework			
Methodological orientation and Theory	9	What methodological orientation was stated to underpin the study? e.g. grounded theory, discourse analysis, ethnography, phenomenology, content analysis	4-8
Participant selection			
Sampling	10	How were participants selected? e.g. purposive, convenience, consecutive, snowball	5
Method of approach	11	How were participants approached? e.g. face-to-face, telephone, mail, email	-
Sample size	12	How many participants were in the study?	1, 5
Non-participation	13	How many people refused to participate or dropped out? Reasons?	5
Setting			
Setting of data collection	14	Where was the data collected? e.g. home, clinic, workplace	1, 6
Presence of non-participants	15	Was anyone else present besides the participants and researchers?	5-6
Description of sample	16	What are the important characteristics of the sample? e.g. demographic data, date	5, 10
Data collection			
Interview guide	17	Were questions, prompts, guides provided by the authors? Was it pilot tested?	6-8
Repeat interviews	18	Were repeat inter views carried out? If yes, how many?	-
Audio/visual recording	19	Did the research use audio or visual recording to collect the data?	6
Field notes	20	Were field notes made during and/or after the inter view or focus group?	8
Duration	21	What was the duration of the inter views or focus group?	6
Data saturation	22	Was data saturation discussed?	-
Transcripts returned	23	Were transcripts returned to participants for comment and/or	7-9

For peer review only

Topic	Item No.	Guide Questions/Description	Reported on Page No.
		correction?	
**Domain 3: analysis and findings**			
Data analysis			
Number of data coders	24	How many data coders coded the data?	6
Description of the coding tree	25	Did authors provide a description of the coding tree?	-
Derivation of themes	26	Were themes identified in advance or derived from the data?	6-8
Software	27	What software, if applicable, was used to manage the data?	6
Participant checking	28	Did participants provide feedback on the findings?	7-9
Reporting			
Quotations presented	29	Were participant quotations presented to illustrate the themes/findings? Was each quotation identified? e.g. participant number	10-20
Data and findings consistent	30	Was there consistency between the data presented and the findings?	10-20
Clarity of major themes	31	Were major themes clearly presented in the findings?	10-20
Clarity of minor themes	32	Is there a description of diverse cases or discussion of minor themes?	10-20

Developed from: Tong A, Sainsbury P, Craig J. Consolidated criteria for reporting qualitative research (COREQ): a 32-item checklist for interviews and focus groups. *International Journal for Quality in Health Care*. 2007. Volume 19, Number 6: pp. 349 – 357

Once you have completed this checklist, please save a copy and upload it as part of your submission. DO NOT include this checklist as part of the main manuscript document. It must be uploaded as a separate file.

For peer review only
